# Supplementary material for: Temperature-dependent Small RNA Expression Depends on Wild Genetic Backgrounds of Caenorhabditis briggsae
Source: Mol Biol Evol. 2022 Oct 12;39(11):msac218. doi: 10.1093/molbev/msac218 (PMC9641977; doi:10.1093/molbev/msac218)
Supplement: msac218_Supplementary_Data [file msac218_supplementary_data.zip › Supplementary Information.pdf]

Supplementary Tables

Table S1: Proportion of 100kb bins along chromosome arm domains where mean 22G-RNA expression was lower at 30ºC than it was at 20ºC, for both genotypes. Bins showing decreased expression at 30ºC were used to calculate the mean magnitude of the expression decrease for both genotypes. Expression decreases are in units of $\log_2$ Reads Per Million.

**Chromosome**	**% of bins in AF16 with decreased expression at 30** ºC	**% of bins in HK104 with decreased expression at 30** ºC	**Average expression decrease for AF16 bins ($\log_2$ RPM)**	**Average expression decrease for HK104 bins ($\log_2$ RPM)**
I	64% (58 / 90)	84% (76 / 90)	0.55	1.33
II	70% (68 / 96)	88% (84 / 96)	0.72	1.60
III	62% (53 / 86)	86% (74 / 86)	0.57	1.33
IV	71% (67 / 95)	80% (76 / 95)	0.73	1.35
V	69% (80 / 116)	77% (89 / 116)	0.63	1.25
X	76% (112 / 147)	82% (120 / 147)	0.74	1.82

Table S2: Proportion of 100kb bins along chromosome center domains where mean 22G-RNA expression was lower at 30ºC than it was at 20ºC, for both genotypes. Bins showing decreased expression at 30ºC were used to calculate the mean magnitude of the expression decrease for both genotypes. Expression decreases are in units of $\log_2$ Reads Per Million.

**Chromosome**	**% of bins in AF16 with decreased expression at 30** °C	**% of bins in HK104 with decreased expression at 30** °C	**Average expression decrease for AF16 bins ($\log_2$ RPM)**	**Average expression decrease for HK104 bins ($\log_2$ RPM)**
I	64% (42 / 66)	92% (61 / 66)	0.46	1.49
II	55% (39 / 71)	93% (66 / 71)	0.42	1.43
III	39% (24 / 61)	90% (55 / 61)	0.31	1.42
IV	57% (46 / 81)	85% (69 / 81)	0.45	1.34
V	54% (43 / 80)	89% (71 / 80)	0.43	1.09
X	81% (57 / 70)	91% (64 / 70)	0.74	1.89

Table S3: voom-normalized expression of 22G-RNAs aligning antisense to 14,283 genomic features in all 17 replicates, in units of $\log_2$ RPM. “Gene_Biotype” indicates the type of feature (protein-coding gene, pseudogene, repeat, or transposable element). AF and HK refer to the AF16 and HK104 strains, respectively. 14, 20, and 30 indicate the rearing temperature of each replicate. “Differential_Expression_Category” indicates which category of differential expression each feature was assigned to.

Table S4: Targets of 22G-RNA pathway proteins in *C. briggsae* used in this study. CSR-1 targets are known for *C. briggsae*; targets of all other proteins are orthologs of known targets in *C. elegans*. Genes with available 22G-RNA or mRNA expression data are also indicated. “TRUE” indicates that a gene belongs to a given set of genes.

Table S5. Number of reads present in each replicate at various stages of the alignment and counting pipeline. AF and HK refer to the AF16 and HK104 strains, respectively. 14, 20, and 30 indicate the rearing temperature of each replicate. Statistics are for alignments to the respective genome (AF samples to the AF16 genome, and HK samples to the HK104 genome).

Replicate	Total # of raw reads	# of reads after trimming	# of reads aligned to the genome	# of reads aligned perfectly to the genome	# of perfectly-aligned reads aligned to an annotated feature
AF14-1	9,714,767	8,624,894	7,596,951	6,664,095	5,048,645
AF14-2	10,057,606	9,369,252	8,663,343	7,419,685	5,292,509
AF14-3	9,153,959	8,341,891	7,837,217	6,934,035	5,942,667
AF20-1	8,311,109	7,021,982	6,613,825	5,912,723	4,929,868
AF20-2	7,187,732	6,047,269	5,738,135	5,153,967	4,240,473
AF20-3	9,901,647	8,922,678	8,122,788	7,109,900	5,562,831
AF30-1	21,556,148	20,809,661	18,949,949	16,009,149	11,746,185
AF30-2	5,557,795	4,641,808	4,317,344	3,815,140	2,887,224
AF30-3	13,334,748	11,638,513	10,736,202	9,141,443	7,399,551
HK14-1	12,155,046	8,868,287	7,663,612	6,503,096	4,493,860
HK14-2	11,165,762	9,530,022	8,239,591	6,958,250	4,845,744
HK14-3	10,256,635	9,393,639	8,205,938	7,011,489	4,913,968
HK20-1	11,667,443	10,624,773	9,621,489	8,498,648	6,082,637
HK20-2	13,321,345	12,516,817	11,097,900	9,592,262	6,639,486
HK20-3	11,495,618	10,514,634	9,462,109	8,163,107	6,094,041
HK30-1	5,928,373	1,298,689	1,016,104	888,207	551,310
HK30-2	19,716,033	15,924,990	13,997,848	12,362,971	7,983,884
HK30-3	12,047,216	9,796,474	7,629,207	6,631,820	3,998,095

Table S6. Alignment statistics for all six genotype-temperature combinations. Small RNAs from each replicate were aligned to both the AF16 reference genome and HK104 pseudo-reference genome. Statistics for each replicate were averaged out within each genotype-temperature combination, excluding the outlier HK30-1 replicate.

Condition	Proportion of reads aligning uniquely to AF16 genome	Proportion of reads aligning uniquely to HK104 genome	Mismatch rate per base against AF16 genome	Mismatch rate per base against HK104 genome
AF16, 14°C	74.0%	72.7%	0.22%	0.34%
AF16, 20°C	73.9%	71.5%	0.17%	0.34%
AF16, 30°C	70.7%	69.1%	0.21%	0.34%
HK104, 14°C	65.6%	67.2%	0.43%	0.35%
HK104, 20°C	68.8%	70.4%	0.36%	0.29%
HK104, 30°C	67.8%	68.9%	0.25%	0.20%

Supplementary Figure Legends

Figure S1. Mean expression of small RNAs mapping to different classes of genomic features in the *C. briggsae* genome, separated by the 5' nucleotide of mapped small RNAs. "AS" indicates reads mapping antisense to a feature.

Figure S2. Total expression of 21U-RNAs aligning to piRNA loci along the length of chromosome I using genomic intervals 150kb wide, in AF16 (top) and HK104 (bottom) worms, considering only piRNA loci present in the genome annotations for both strains. Expression is in units of log2 Reads Per Million. Shaded regions give ±1 standard deviation around the mean for each set of replicates.

Figure S3. Multidimensional scaling plot (A) and correlation heatmap (B) of the 22G-RNA expression profiles of 14,283 *C. briggsae* genomic features across all 17 samples. Expression values were normalized to units of $\log_2$ Reads Per Million. Correlations were calculated using Spearman's rank correlation.

Figure S4. Expression of aligned 22G-RNAs across the full length of chromosomes I (A), II (B), III (C), IV (D), and V (E) in AF16 (top) and HK104 (bottom) worms, at each temperature. Expression values were averaged between replicates, using genomic intervals 150 kilobases wide. Expression is in units of log2 Reads Per Million (RPM). Shaded areas extend ±1 standard deviation around the mean expression of each interval.

Figure S5. 22G-RNA expression profiles across all 17 samples, for each of the 5 categories of genomic features defined based on 22G-RNA expression effects (A – E). Features include protein-coding genes, pseudogenes, repeats, and transposons. Expression values for each feature are scaled using row normalization. Rows in each heatmap are ordered based on hierarchical clustering.

Figure S6. Distribution of protein-coding genes showing a genotype-temperature interaction on 22G-RNA expression across the length of each *C. briggsae* chromosome, compared to the genomic distribution of all *C. briggsae* protein-coding genes.

Figure S7. Correlation between 22G-RNA and mRNA expression for all 6 genotype-temperature combinations. Correlations were calculated using the set of all 12,623 genes (top row), as well as using only the genes in each genotype-temperature combination that were in the top 10% of 22G-RNA expression (bottom row). Expression values were normalized to units of $\log_2$ Reads Per Million. Correlations were calculated using Spearman's rank correlation. The positive correlation seen when using all genes is weakest in temperate HK104 worms reared under both hot and cold chronic thermal stress (HK104 at 14ºC Spearman's $\rho = 0.28$; HK104 at 30ºC Spearman's $\rho = 0.23$; all other treatment Spearman's ρ ranges from 0.38 to 0.54). Compared to 20ºC, the correlation in HK104 is significantly lower at both 14ºC (*z*-test, $p < 2.2 \times 10^{-16}$) and 30ºC ($p < 2.2 \times 10^{-16}$). In AF16 worms, the correlation is also lower at 14ºC (*z*-test, $p < 2.2 \times 10^{-16}$) but higher at 30ºC ($p = 6.7 \times 10^{-13}$), compared to 20ºC. The negative correlation seen when using the genes in the top 10% of 22G-RNA expression is weaker in HK104 at extreme temperatures compared to all other genotype-temperature combinations (HK104 at 14ºC Spearman's $\rho = -0.32$; HK104 at 30ºC Spearman's $\rho = -0.22$; all other treatment Spearman's ρ ranges from -0.36 to -0.41). Whereas HK104 worms showed a significantly weaker correlation at

both 14ºC (*z*-test, $p = 0.021$) and 30ºC ($p = 5.9 \times 10^{-7}$) relative to 20ºC, AF16 worms did not show significantly different correlations at either 14ºC ($p = 0.260$) or 30ºC ($p = 0.602$) compared to 20ºC.

Figure S8. 22G-RNA and mRNA expression for each genotype-temperature combination, for 12,623 genes with 22G-RNA and mRNA expression data available. A quadratic curve of best fit is also shown, with the shaded area around the curve representing the standard error. Colors indicate whether each gene is a target of CSR-1, WAGO-1, both, or neither. As we observed for genes overall, genes targeted by CSR-1 and WAGO-1 both showed a significant positive correlation between 22G-RNA and mRNA expression (CSR-1 target Spearman's ρ ranges from 0.10 to 0.40 with $p < 2.5 \times 10^{-9}$ in all genotype-temperature combinations; WAGO-1 target Spearman's ρ ranges from 0.15 to 0.36 with $p < 2.3 \times 10^{-5}$ in all genotype-temperature combinations). Among genes in the top 10% of 22G-RNA expression for WAGO-1 or CSR-1 targets, WAGO-1 targets tended to have a more negative 22G-RNA : mRNA correlation than CSR-1 targets, consistent with the silencing role of WAGO-1. In particular, at 20ºC, the WAGO-1 22G-RNA : mRNA correlation for these genes was significantly negative in HK104 worms but not in AF16 worms (HK104 Spearman's $\rho = -0.29$, $p = 0.0076$; AF16 $\rho = -0.15$, $p = 0.160$), whereas CSR-1 targets did not show a significant correlation in either HK104 ($\rho = -0.02$, $p = 0.682$) or AF16 ($\rho = -0.04$, $p = 0.457$) animals at this temperature. We observed qualitatively similar but non-significant trends at the more extreme rearing temperatures, indicating that temperature extremes weaken the association between 22G-RNA expression and the expression levels of their targets (WAGO-1 Spearman's ρ: -0.05 to -0.21 with $p > 0.056$; CSR-1 Spearman's ρ: 0.06 to -0.07 with $p > 0.14$).

Figure S9. Overlap between genes showing a given effect on 22G-RNA expression, and genes showing the same effect on mRNA expression, for different categories of expression effects (A – E).

Figure S10. mRNA expression of different genes involved in the 26G-RNA and piRNA pathways, across genotypes and temperatures. *CBG09950* is the *C. briggsae* ortholog of both *alg-3* and *alg-4*. Error bars give ±1 standard deviation around the mean for each set of replicates. Dashed lines connect mean expression values for each genotype across temperatures. Linear modeling was used to determine the significance of genotype-temperature interactions between 14ºC and 20ºC, and between 20ºC and 30ºC, with * indicating $p < 0.05$, ** indicating $p < 0.01$, *** indicating $p < 0.001$, and n.s. indicating non-significance.

Figure S11. Overlap between the genes targeted by WAGO-1 or RRF-1 (A), between the genes targeted by CSR-1 or EGO-1 (B), and between the genes targeted by CSR-1, WAGO-1, or HRDE-1 (C).

Figure S12. Distribution of mRNA expression values in each genotype-temperature combination, for genes that are targeted by CSR-1 but not WAGO-1 (4524 genes), both CSR-1 and WAGO-1 simultaneously (112 genes), or WAGO-1 but not CSR-1 (906 genes). Significant differences between sets of genes were determined using a Wilcoxon rank sum test, with *** indicating $p < 0.001$ and n.s. indicating non-significance.

Figure S13. 22G-RNA expression of all genes targeted by CSR-1a or CSR-1b (A) as well as genes exclusively targeted by only one CSR-1 isoform and not the other (B), for each genotype-temperature combination. Significant differences between temperature treatments of the same

genotype were determined using a Wilcoxon rank sum test, with * indicating $p < 0.05$, ** indicating $p < 0.01$, *** indicating $p < 0.001$, and n.s. indicating non-significance.

Figure S14. 22G-RNA expression of genes targeted by HRDE-1 for each genotype-temperature combination, separated based on whether or not the gene is also targeted by EGO-1. Significant differences between temperature treatments of the same genotype were determined using a Wilcoxon rank sum test, with * indicating $p < 0.05$, *** indicating $p < 0.001$, and n.s. indicating non-significance.

Figure S15. mRNA expression of genes targeted by EGO-1, RRF-1, CSR-1, WAGO-1, HRDE-1, CSR-1a, or CSR-1b, as well as all genes showing 22G-RNA expression, for each genotype-temperature combination. Significant differences between temperature treatments of the same genotype were determined using a Wilcoxon rank sum test, with * indicating $p < 0.05$, ** indicating $p < 0.01$, *** indicating $p < 0.001$, and n.s. indicating non-significance.

Figure S16. 22G-RNA expression of genes targeted by EGO-1, RRF-1, CSR-1, WAGO-1, or HRDE-1, as well as all genes showing 22G-RNA expression, for each genotype-temperature combination, using only genes classified as having "flat" gene expression across *C. elegans* development. Significant differences between temperature treatments of the same genotype were determined using a Wilcoxon rank sum test, with * indicating $p < 0.05$, ** indicating $p < 0.01$, *** indicating $p < 0.001$, and n.s. indicating non-significance.

Figure S17. 22G-RNA expression of genes targeted by EGO-1, RRF-1, CSR-1 (including both CSR-1a and CSR-1b), WAGO-1, or HRDE-1, as well as all genes showing 22G-RNA expression, for each genotype-temperature combination. 22G-RNA expression values were normalized using the TMM method to meet the assumption that most genes in the genome do not show differential 22G-RNA expression, followed by voom normalization to units of $\log_2$ RPM. Significant differences between temperature treatments of the same genotype were determined using a Wilcoxon rank sum test, with * indicating $p < 0.05$, ** indicating $p < 0.01$, *** indicating $p < 0.001$, and n.s. indicating non-significance.

Supplementary Figures

Figure S1.

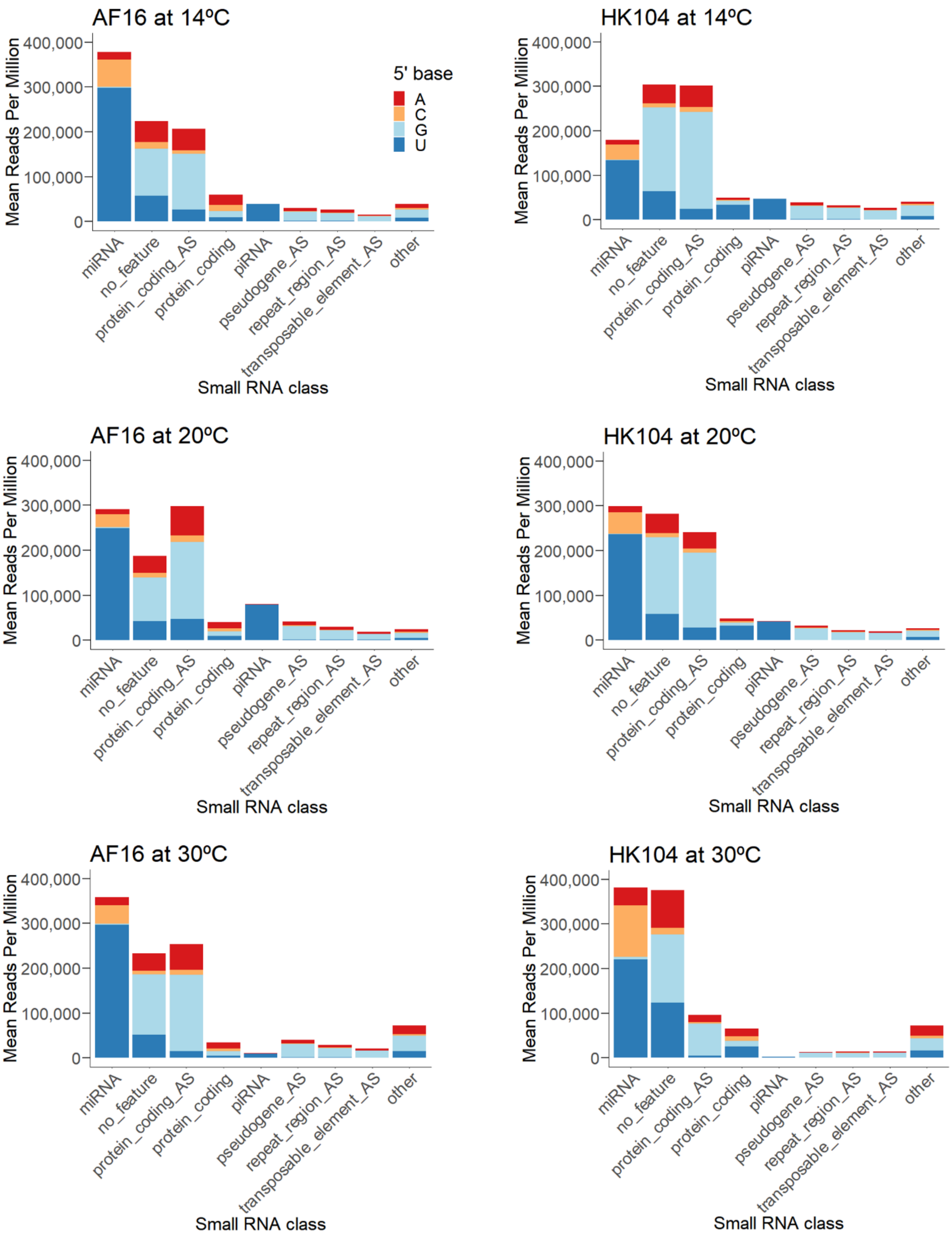

Figure S2.

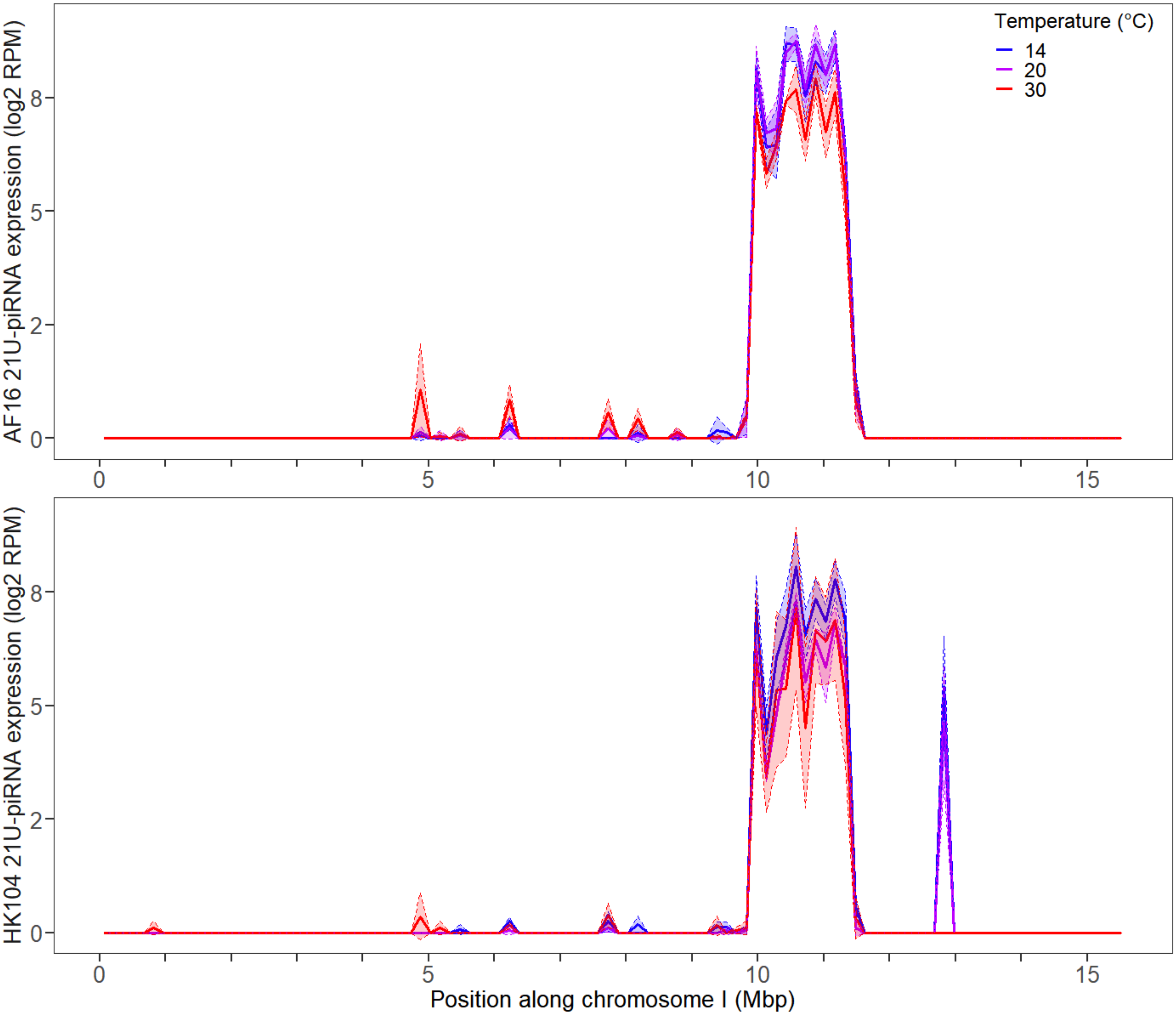

Temperature (°C)
14
20
30
AF16 21U-piRNA expression (log2 RPM)
0
2
5
8
0
5
10
15
HK104 21U-piRNA expression (log2 RPM)
0
2
5
8
0
5
10
15
Position along chromosome I (Mbp)

Figure S3.

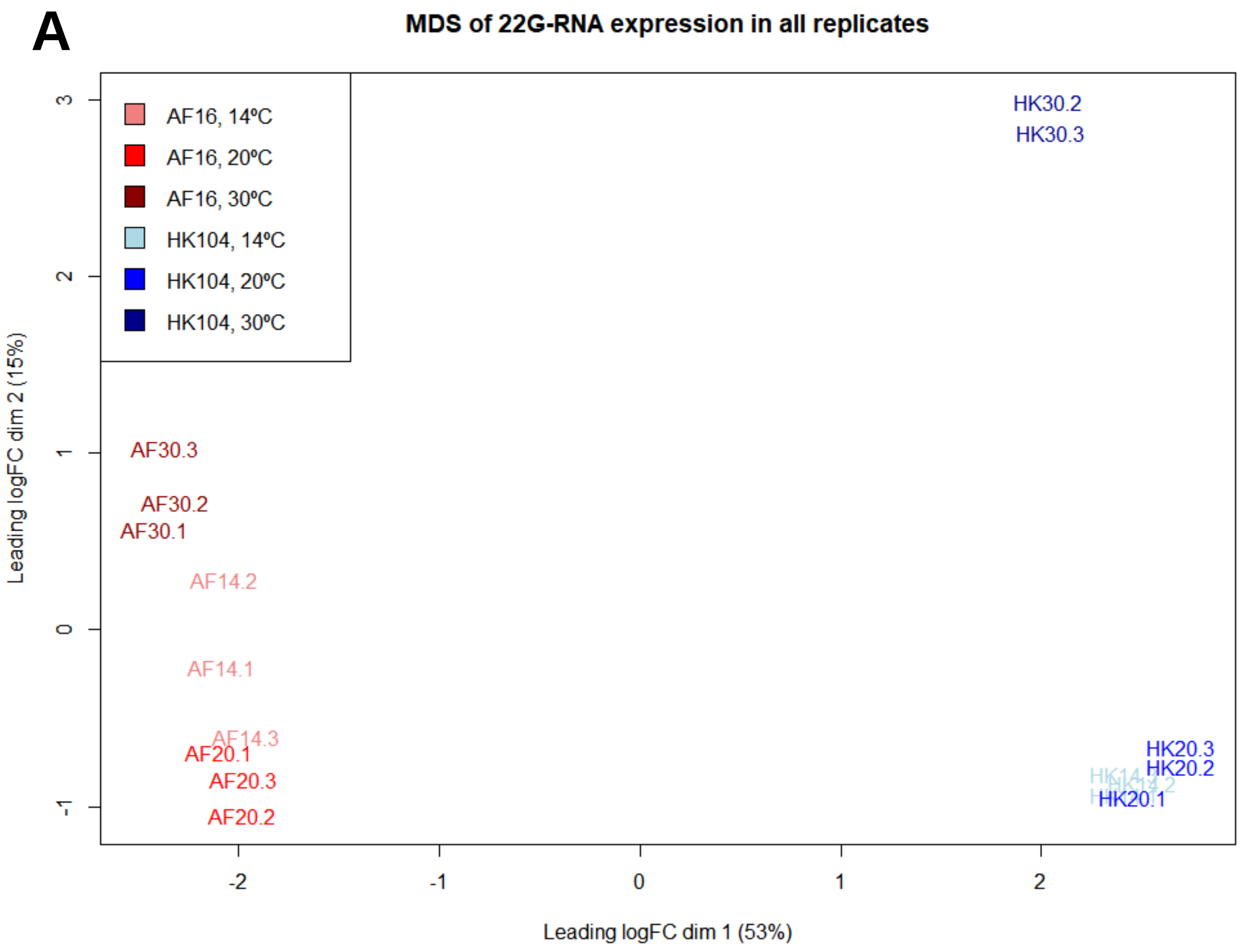
A
MDS of 22G-RNA expression in all replicates
AF16, 14ºC
AF16, 20ºC
AF16, 30ºC
HK104, 14ºC
HK104, 20ºC
HK104, 30ºC
HK30.2
HK30.3
AF30.3
AF30.2
AF30.1
AF14.2
AF14.1
AF14.3
AF20.1
AF20.3
AF20.2
HK20.3
HK20.2
HK20.1
Leading logFC dim 2 (15%)
Leading logFC dim 1 (53%)
-2
-1
0
1
2
3

B

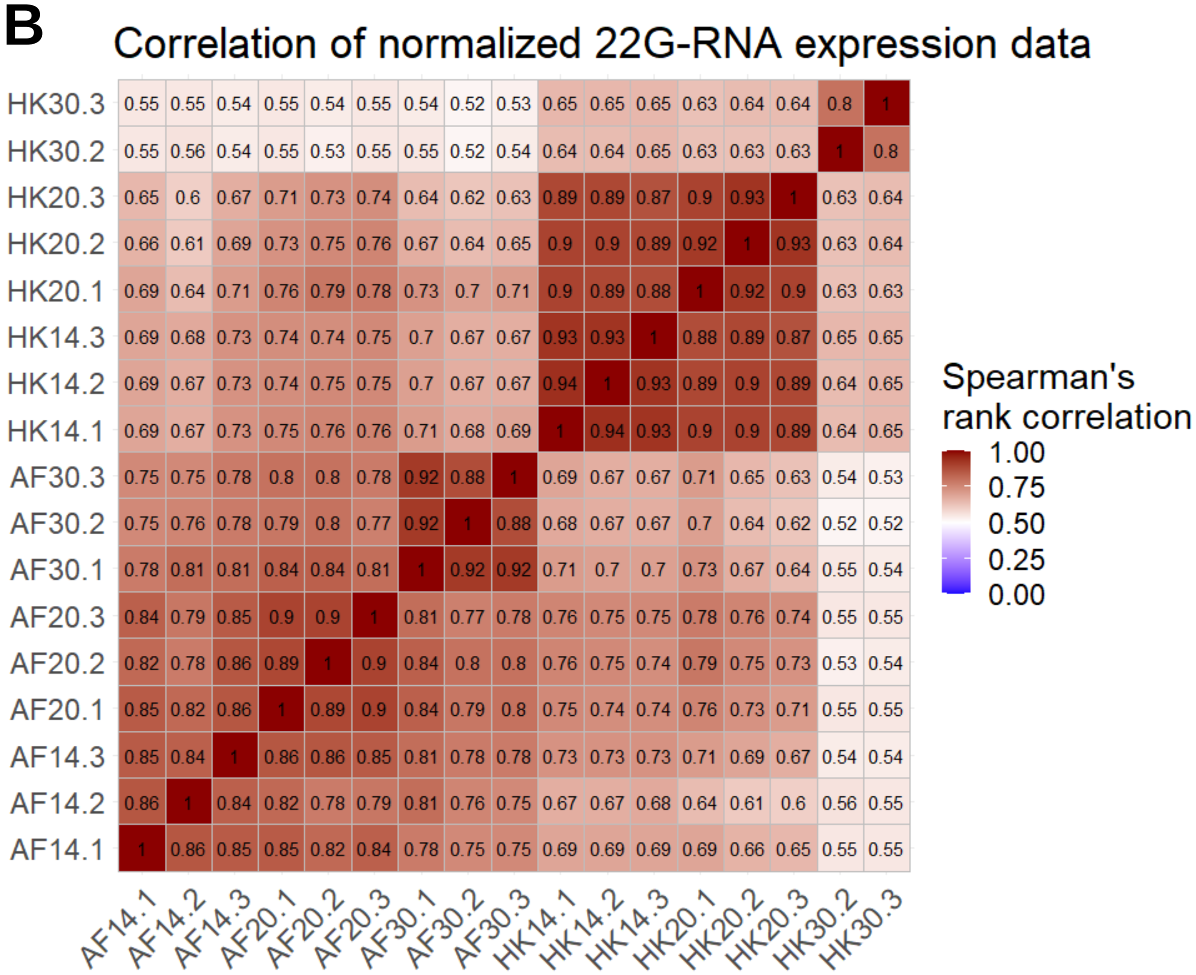
Correlation of normalized 22G-RNA expression data
HK30.3 0.55 0.55 0.54 0.55 0.54 0.55 0.54 0.52 0.53 0.65 0.65 0.65 0.63 0.64 0.64 0.8 1
HK30.2 0.55 0.56 0.54 0.55 0.53 0.55 0.55 0.52 0.54 0.64 0.64 0.65 0.63 0.63 0.63 1 0.8
HK20.3 0.65 0.6 0.67 0.71 0.73 0.74 0.64 0.62 0.63 0.89 0.89 0.87 0.9 0.93 1 0.63 0.64
HK20.2 0.66 0.61 0.69 0.73 0.75 0.76 0.67 0.64 0.65 0.9 0.9 0.89 0.92 1 0.93 0.63 0.64
HK20.1 0.69 0.64 0.71 0.76 0.79 0.78 0.73 0.7 0.71 0.9 0.89 0.88 1 0.92 0.9 0.63 0.63
HK14.3 0.69 0.68 0.73 0.74 0.74 0.75 0.7 0.67 0.67 0.93 0.93 1 0.88 0.89 0.87 0.65 0.65
HK14.2 0.69 0.67 0.73 0.74 0.75 0.75 0.7 0.67 0.67 0.94 1 0.93 0.89 0.9 0.89 0.64 0.65
HK14.1 0.69 0.67 0.73 0.75 0.76 0.76 0.71 0.68 0.69 1 0.94 0.93 0.9 0.9 0.89 0.64 0.65
AF30.3 0.75 0.75 0.78 0.8 0.8 0.78 0.92 0.88 1 0.69 0.67 0.67 0.71 0.65 0.63 0.54 0.53
AF30.2 0.75 0.76 0.78 0.79 0.8 0.77 0.92 1 0.88 0.68 0.67 0.67 0.7 0.64 0.62 0.52 0.52
AF30.1 0.78 0.81 0.81 0.84 0.84 0.81 1 0.92 0.92 0.71 0.7 0.7 0.73 0.67 0.64 0.55 0.54
AF20.3 0.84 0.79 0.85 0.9 0.9 1 0.81 0.77 0.78 0.76 0.75 0.75 0.78 0.76 0.74 0.55 0.55
AF20.2 0.82 0.78 0.86 0.89 1 0.9 0.84 0.8 0.8 0.76 0.75 0.74 0.79 0.75 0.73 0.53 0.54
AF20.1 0.85 0.82 0.86 1 0.89 0.9 0.84 0.79 0.8 0.75 0.74 0.74 0.76 0.73 0.71 0.55 0.55
AF14.3 0.85 0.84 1 0.86 0.86 0.85 0.81 0.78 0.78 0.73 0.73 0.73 0.71 0.69 0.67 0.54 0.54
AF14.2 0.86 1 0.84 0.82 0.78 0.79 0.81 0.76 0.75 0.67 0.67 0.68 0.64 0.61 0.6 0.56 0.55
AF14.1 1 0.86 0.85 0.85 0.82 0.84 0.78 0.75 0.75 0.69 0.69 0.69 0.69 0.66 0.65 0.55 0.55
AF14.1 AF14.2 AF14.3 AF20.1 AF20.2 AF20.3 AF30.1 AF30.2 AF30.3 HK14.1 HK14.2 HK14.3 HK20.1 HK20.2 HK20.3 HK30.2 HK30.3
Spearman's rank correlation
1.00
0.75
0.50
0.25
0.00

Figure S4.

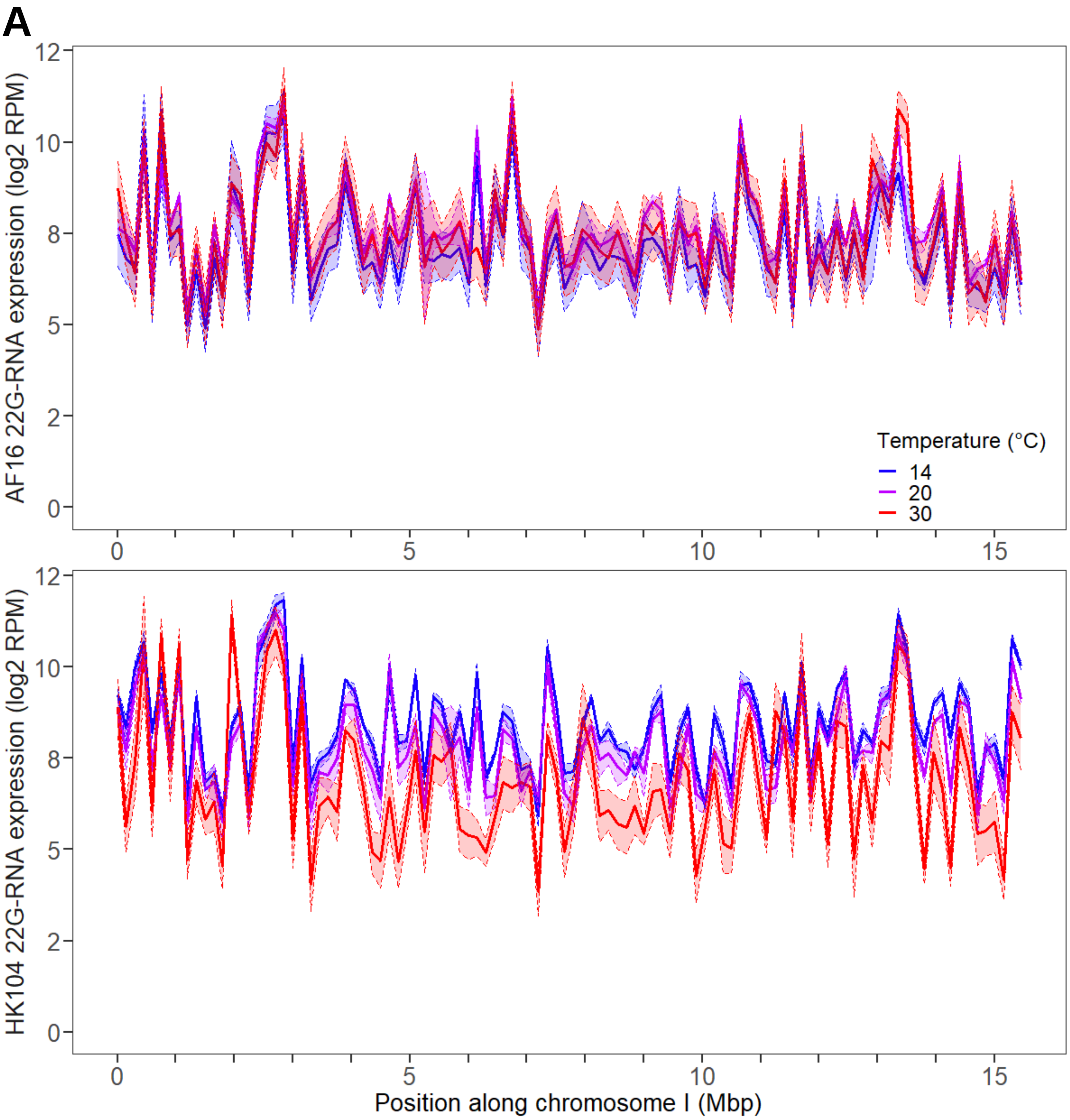
A
AF16 22G-RNA expression (log2 RPM)
12
10
8
5
2
0
Temperature (°C)
14
20
30
0
5
10
15
HK104 22G-RNA expression (log2 RPM)
12
10
8
5
2
0
0
5
10
15
Position along chromosome I (Mbp)

B

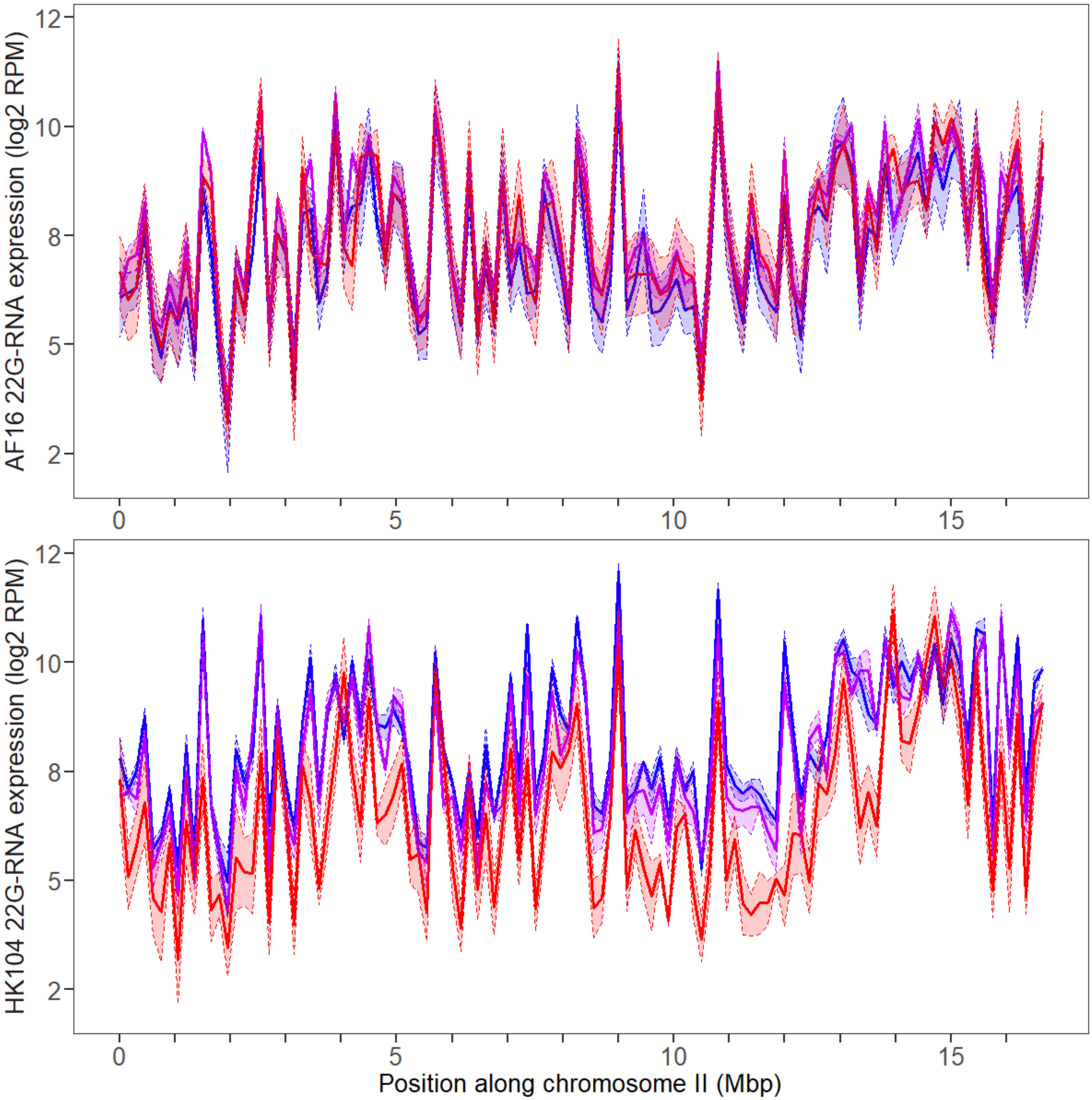
12
10
8
5
2
AF16 22G-RNA expression (log2 RPM)
0
5
10
15
12
10
8
5
2
HK104 22G-RNA expression (log2 RPM)
0
5
10
15
Position along chromosome II (Mbp)

C

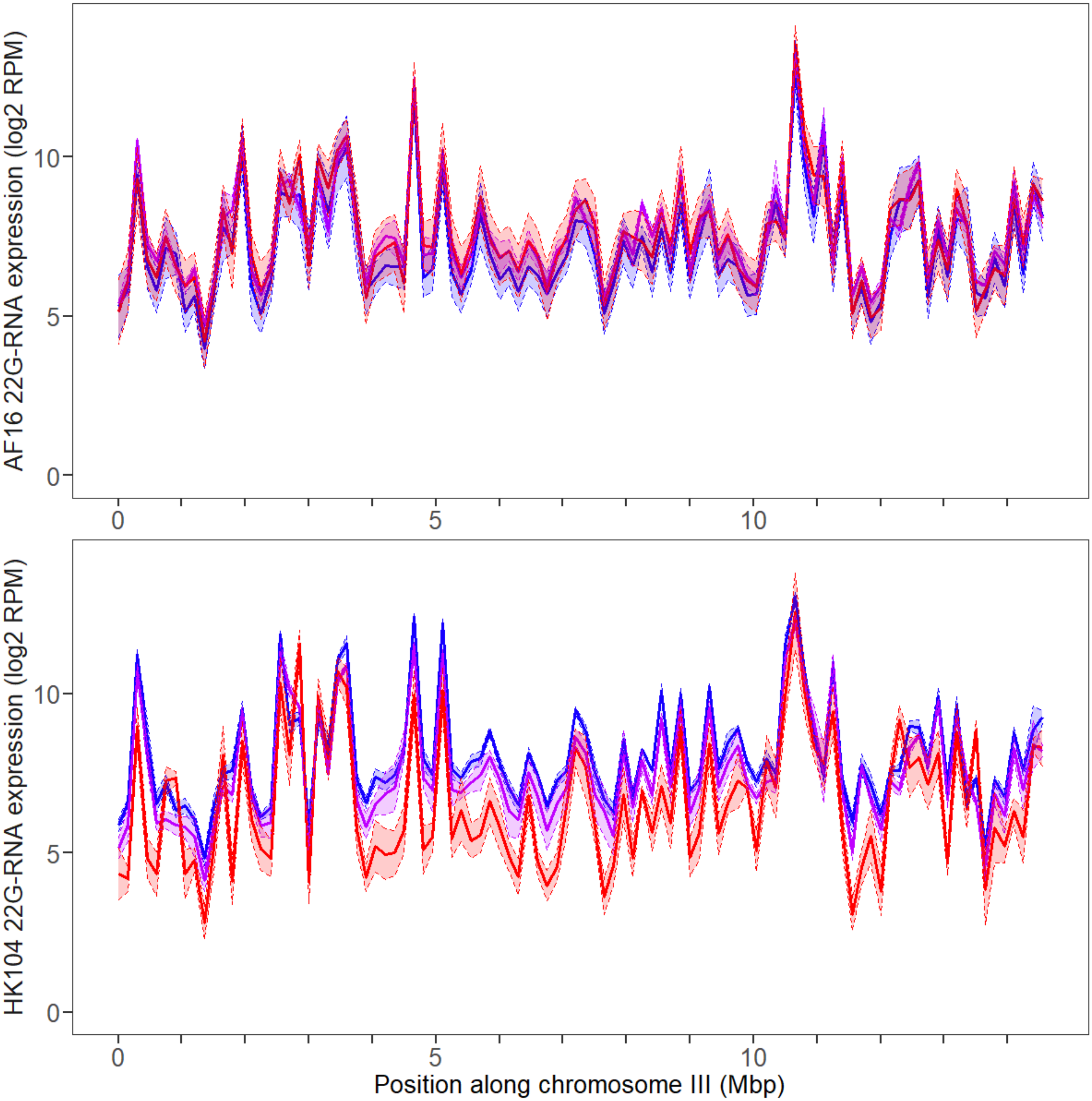
AF16 22G-RNA expression (log2 RPM)
10
5
0
0
5
10
HK104 22G-RNA expression (log2 RPM)
10
5
0
0
5
10
Position along chromosome III (Mbp)

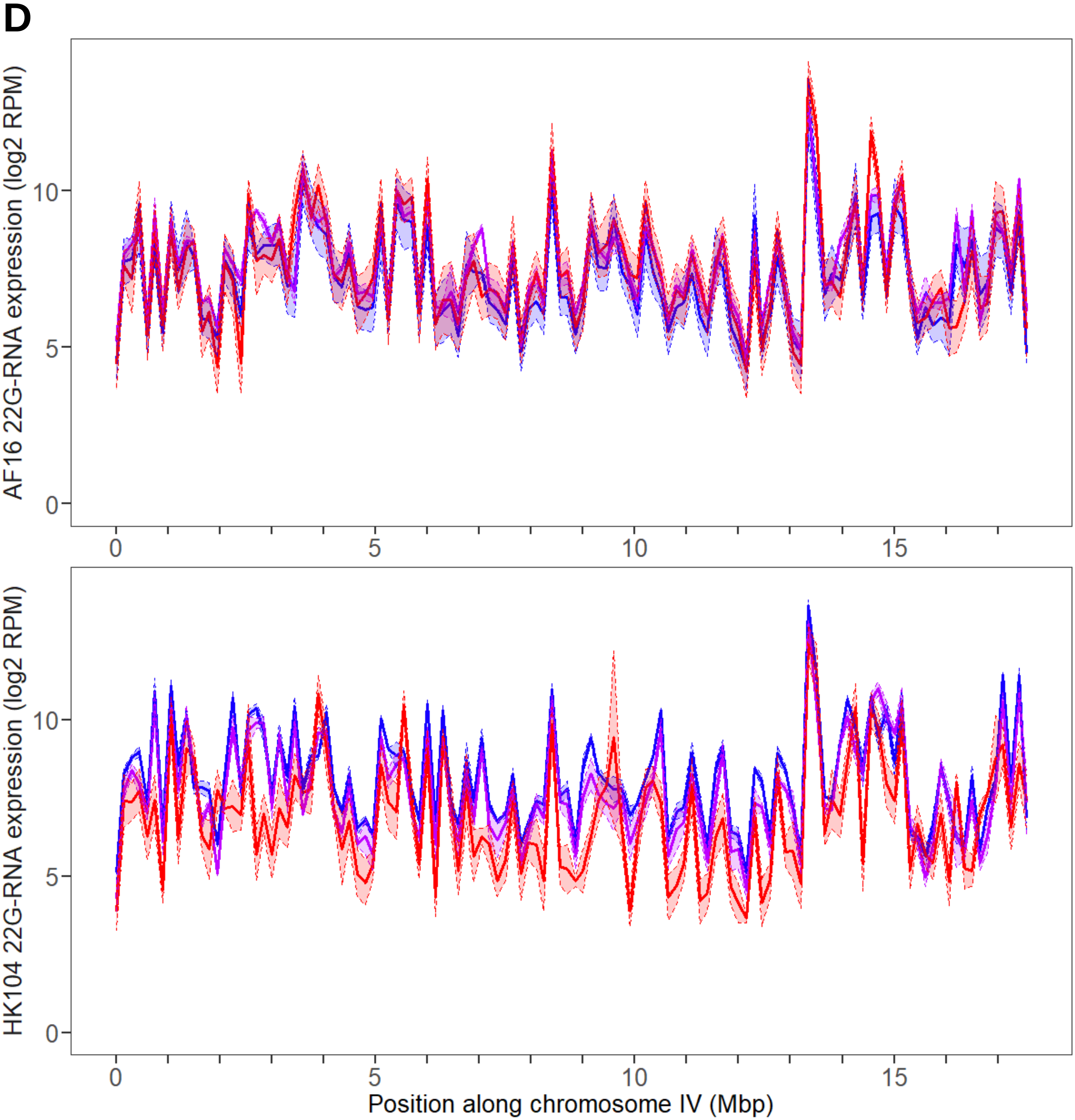
D
AF16 22G-RNA expression (log2 RPM)
0
5
10
0
5
10
15
HK104 22G-RNA expression (log2 RPM)
0
5
10
0
5
10
15
Position along chromosome IV (Mbp)

E

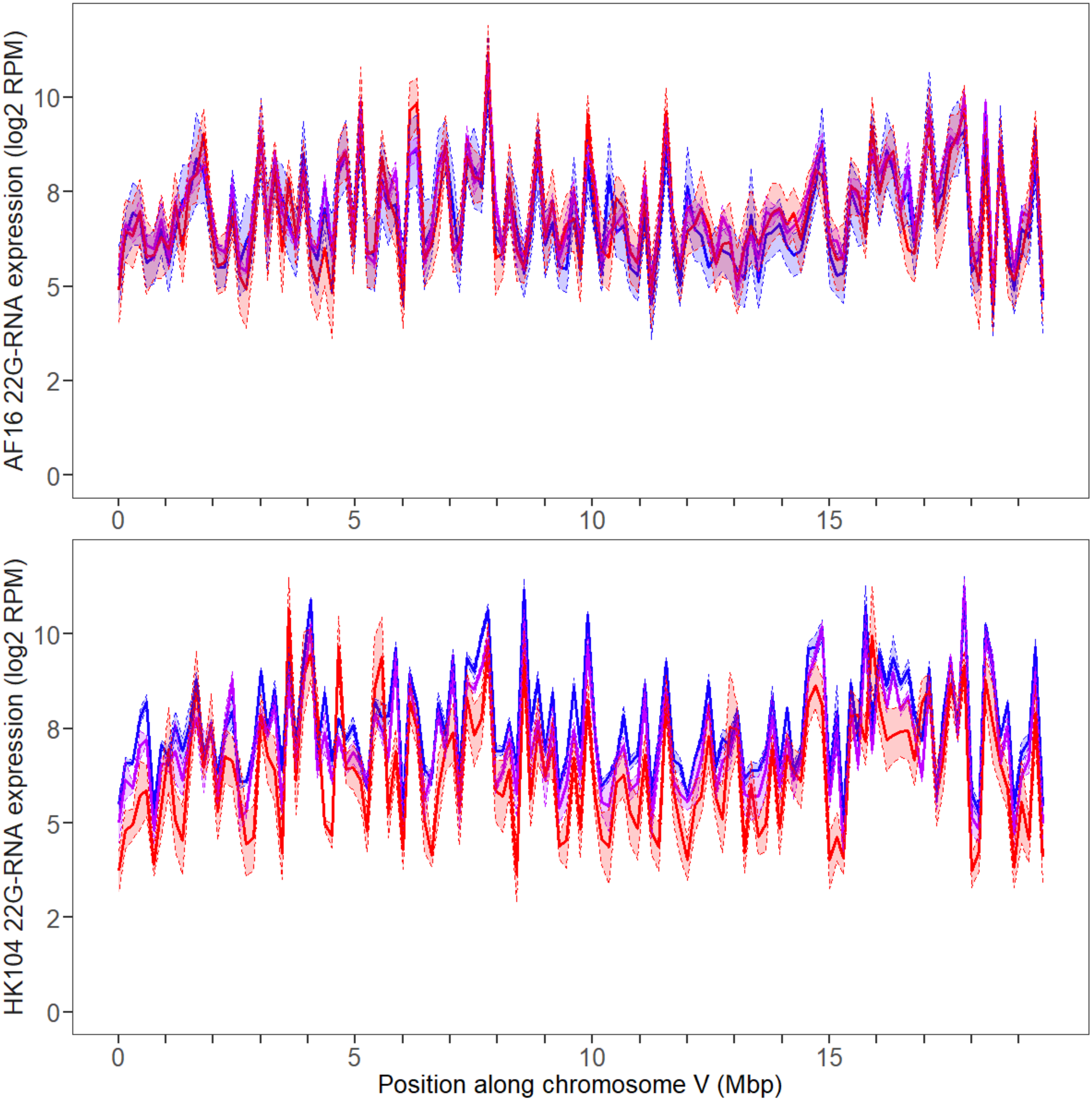
AF16 22G-RNA expression (log2 RPM)
HK104 22G-RNA expression (log2 RPM)
0
2
5
8
10
0
5
10
15
Position along chromosome V (Mbp)

Figure S5.

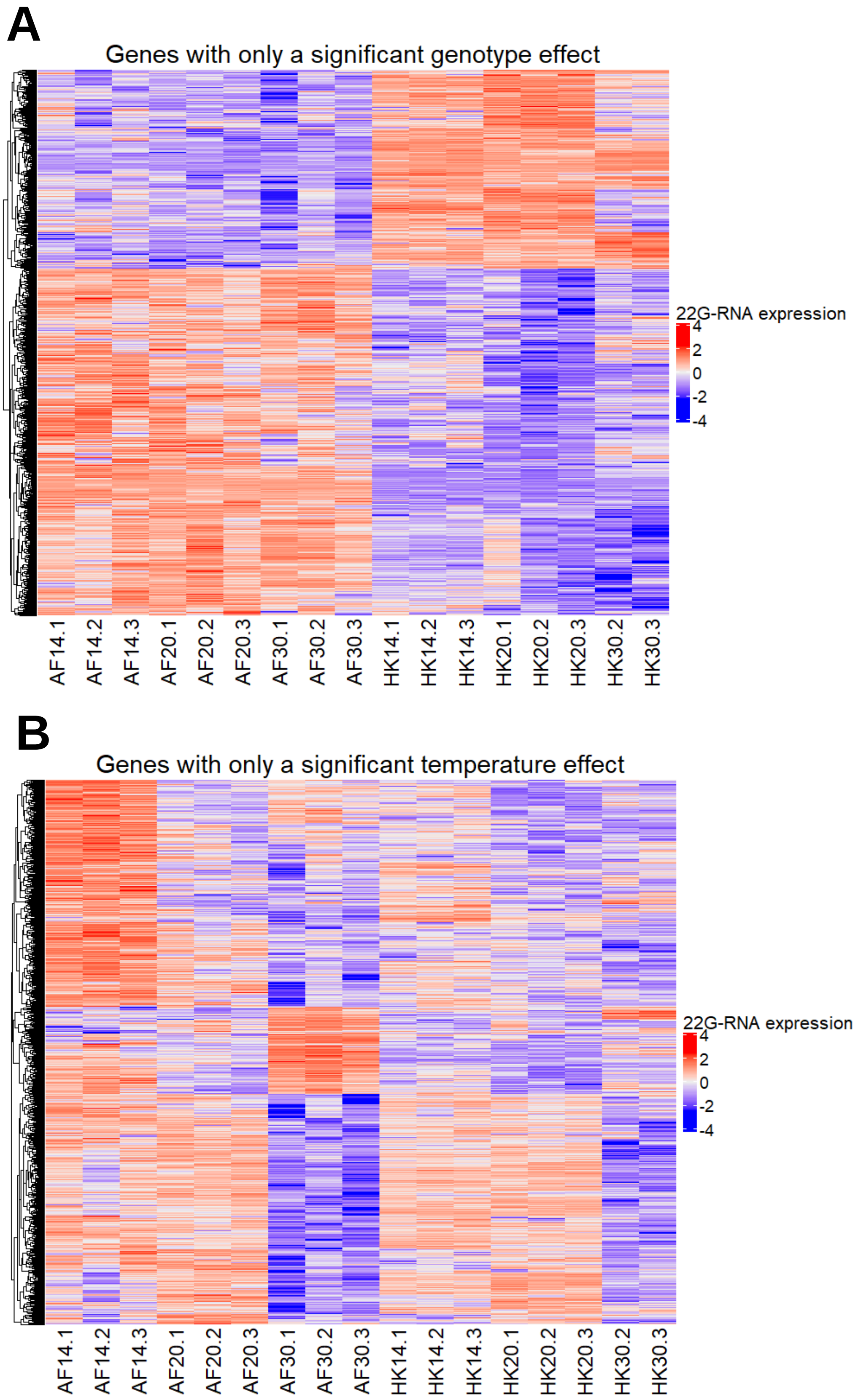
A
Genes with only a significant genotype effect
22G-RNA expression
4
2
0
-2
-4
AF14.1
AF14.2
AF14.3
AF20.1
AF20.2
AF20.3
AF30.1
AF30.2
AF30.3
HK14.1
HK14.2
HK14.3
HK20.1
HK20.2
HK20.3
HK30.2
HK30.3
B
Genes with only a significant temperature effect
22G-RNA expression
4
2
0
-2
-4
AF14.1
AF14.2
AF14.3
AF20.1
AF20.2
AF20.3
AF30.1
AF30.2
AF30.3
HK14.1
HK14.2
HK14.3
HK20.1
HK20.2
HK20.3
HK30.2
HK30.3

C

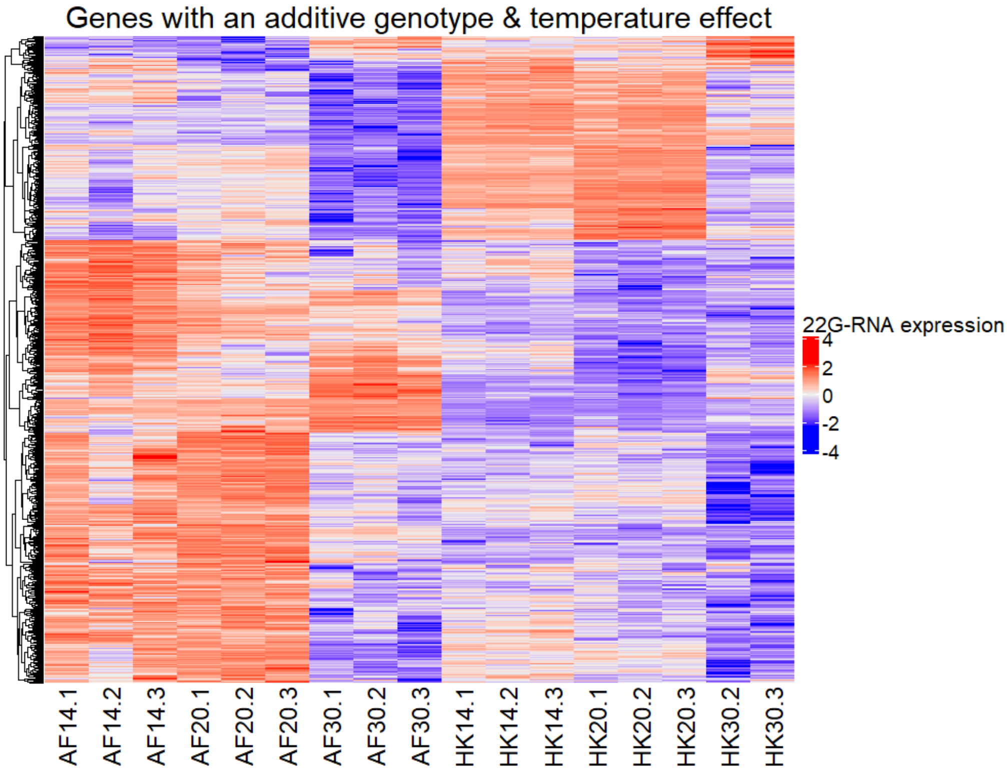
Genes with an additive genotype & temperature effect
22G-RNA expression
4
2
0
-2
-4
AF14.1
AF14.2
AF14.3
AF20.1
AF20.2
AF20.3
AF30.1
AF30.2
AF30.3
HK14.1
HK14.2
HK14.3
HK20.1
HK20.2
HK20.3
HK30.2
HK30.3

D

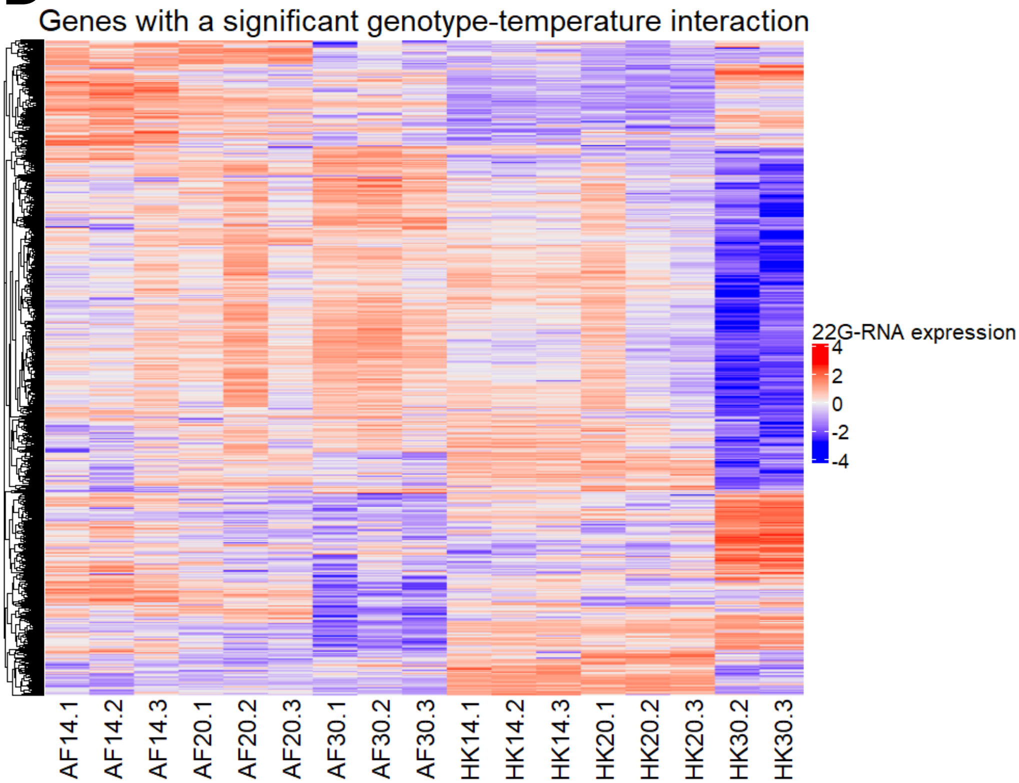
Genes with a significant genotype-temperature interaction
22G-RNA expression
4
2
0
-2
-4
AF14.1
AF14.2
AF14.3
AF20.1
AF20.2
AF20.3
AF30.1
AF30.2
AF30.3
HK14.1
HK14.2
HK14.3
HK20.1
HK20.2
HK20.3
HK30.2
HK30.3

E

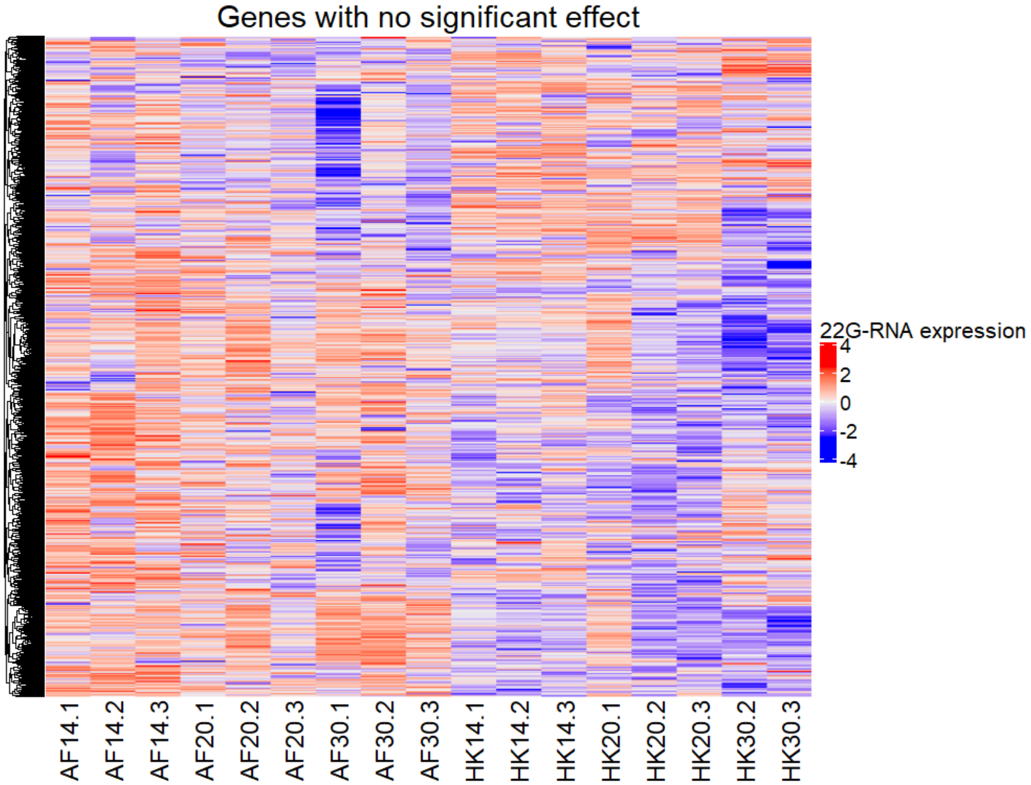

Genes with no significant effect
22G-RNA expression
4
2
0
-2
-4
AF14.1
AF14.2
AF14.3
AF20.1
AF20.2
AF20.3
AF30.1
AF30.2
AF30.3
HK14.1
HK14.2
HK14.3
HK20.1
HK20.2
HK20.3
HK30.2
HK30.3

Figure S6.

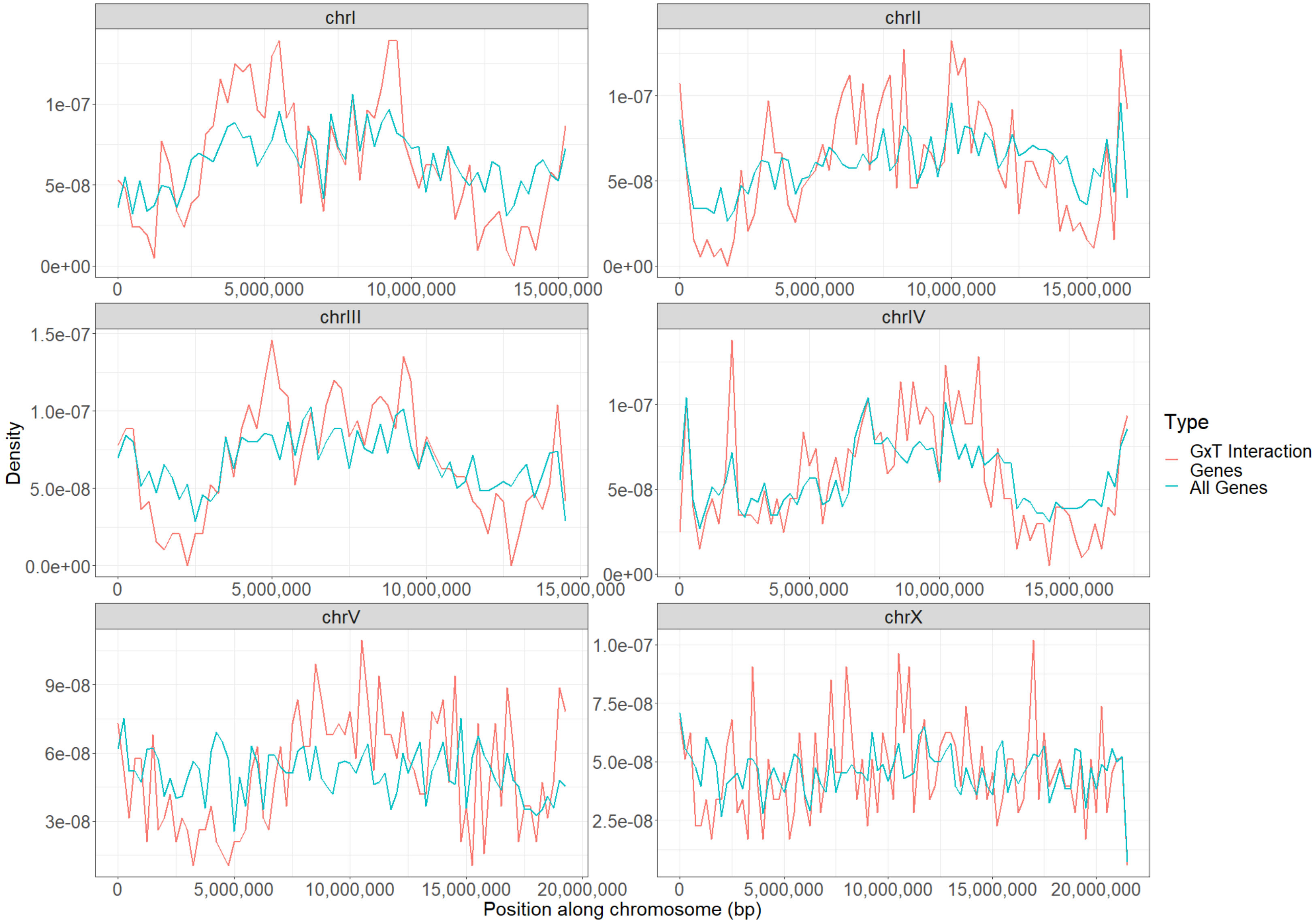

chrI
chrII
chrIII
chrIV
chrV
chrX
1e-07
5e-08
0e+00
1.5e-07
1.0e-07
5.0e-08
0.0e+00
9e-08
6e-08
3e-08
7.5e-08
2.5e-08
0
5,000,000
10,000,000
15,000,000
20,000,000
Density
Position along chromosome (bp)
Type
GxT Interaction Genes
All Genes

Figure S7.

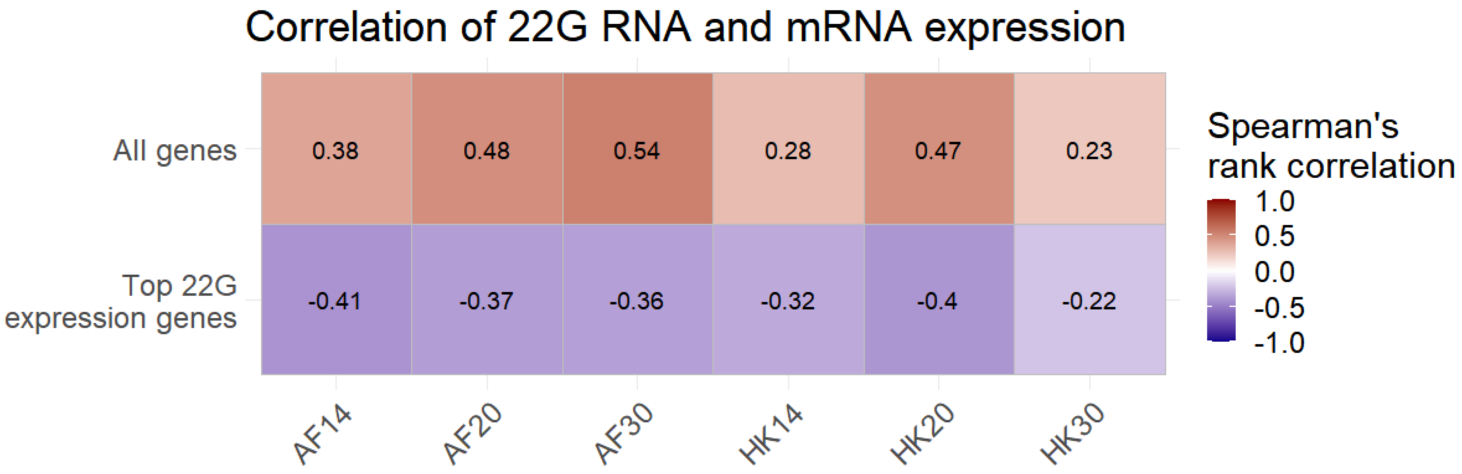

Correlation of 22G RNA and mRNA expression
All genes
Top 22G expression genes
0.38
0.48
0.54
0.28
0.47
0.23
-0.41
-0.37
-0.36
-0.32
-0.4
-0.22
AF14
AF20
AF30
HK14
HK20
HK30
Spearman's rank correlation
1.0
0.5
0.0
-0.5
-1.0

Figure S8.

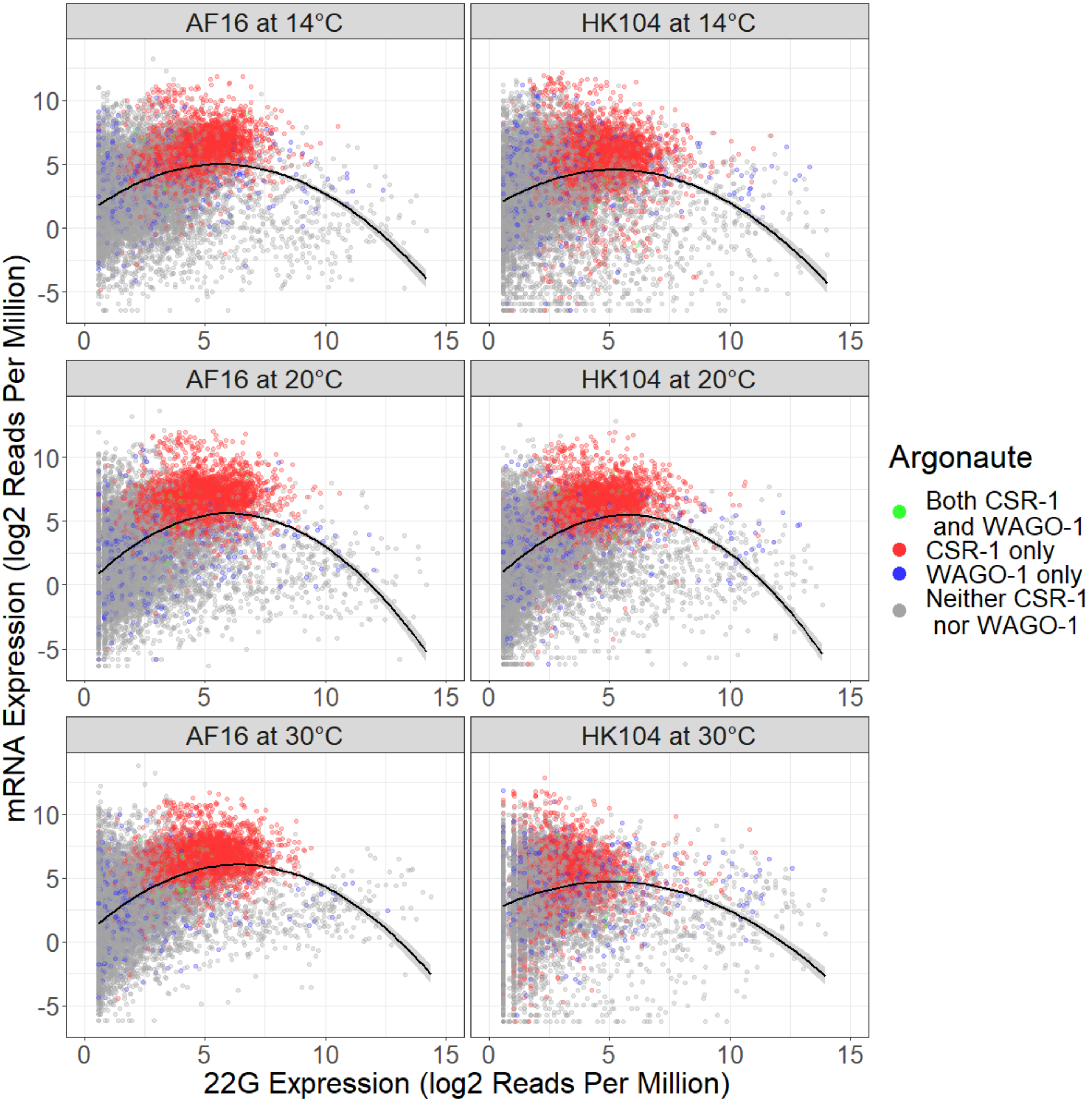

AF16 at 14°C
HK104 at 14°C
AF16 at 20°C
HK104 at 20°C
AF16 at 30°C
HK104 at 30°C
mRNA Expression (log2 Reads Per Million)
22G Expression (log2 Reads Per Million)
Argonaute
Both CSR-1 and WAGO-1
CSR-1 only
WAGO-1 only
Neither CSR-1 nor WAGO-1

Figure S9.

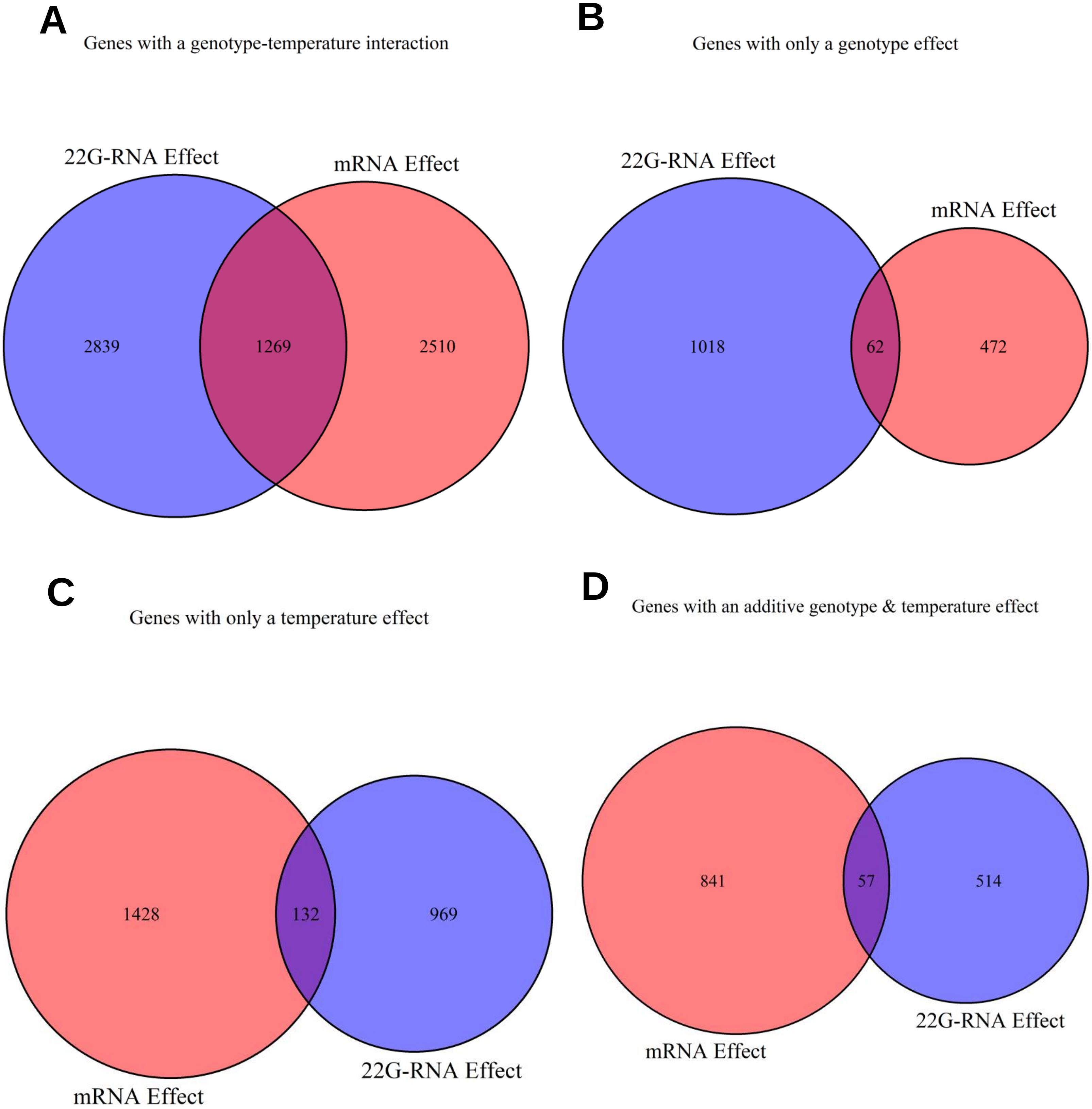
A
Genes with a genotype-temperature interaction
22G-RNA Effect
mRNA Effect
2839
1269
2510
B
Genes with only a genotype effect
22G-RNA Effect
mRNA Effect
1018
62
472
C
Genes with only a temperature effect
1428
132
969
mRNA Effect
22G-RNA Effect
D
Genes with an additive genotype & temperature effect
841
57
514
mRNA Effect
22G-RNA Effect

E

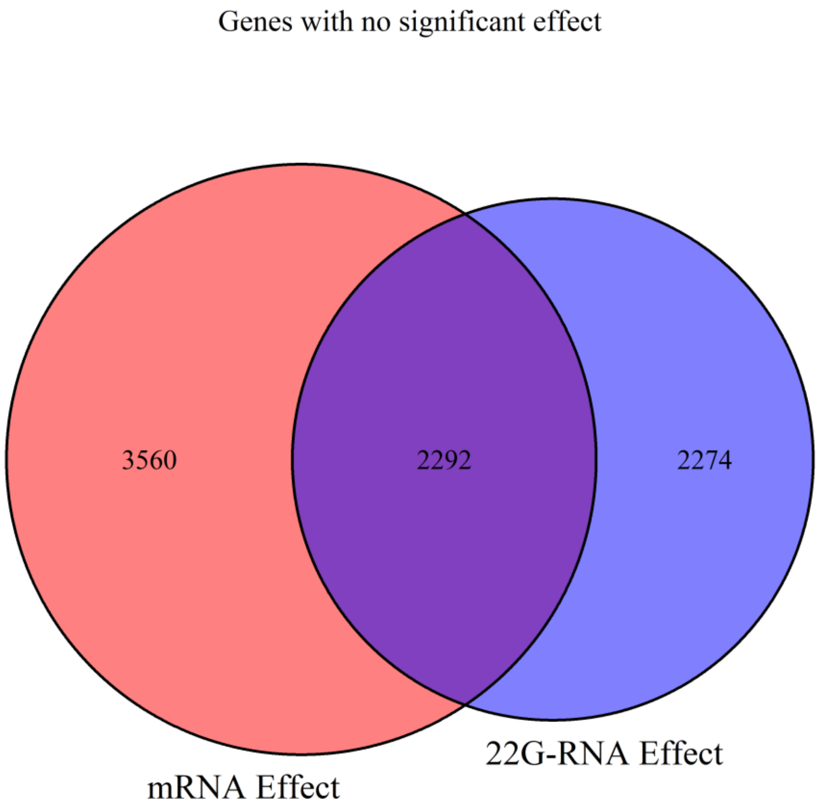
Genes with no significant effect
3560
2292
2274
mRNA Effect
22G-RNA Effect

Figure S10.

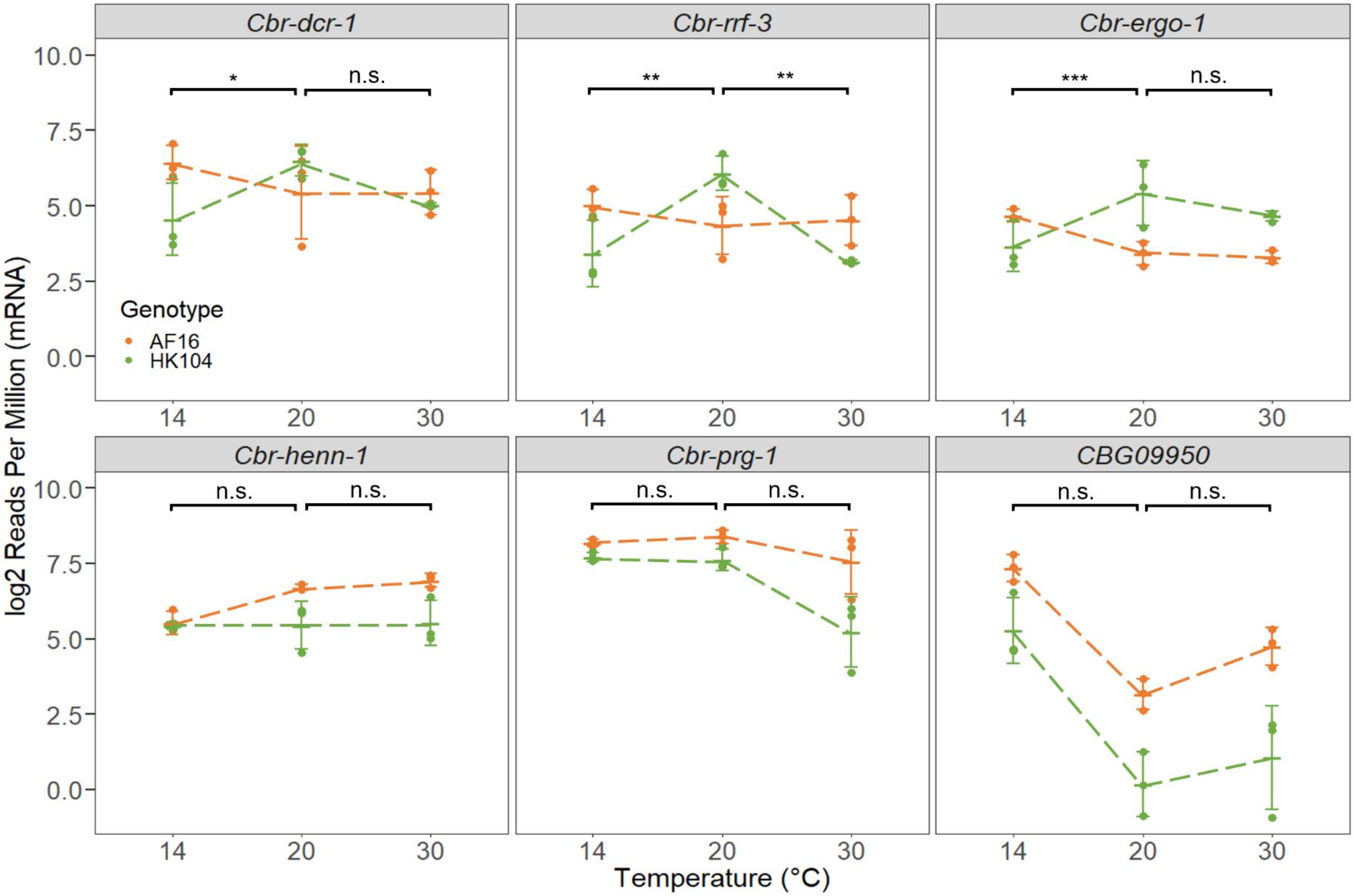

Cbr-dcr-1
Cbr-rrf-3
Cbr-ergo-1
Cbr-henn-1
Cbr-prg-1
CBG09950
*
n.s.
**
**
***
n.s.
n.s.
n.s.
n.s.
n.s.
n.s.
n.s.
Genotype
AF16
HK104
log2 Reads Per Million (mRNA)
10.0
7.5
5.0
2.5
0.0
14
20
30
Temperature (°C)

Figure S11.

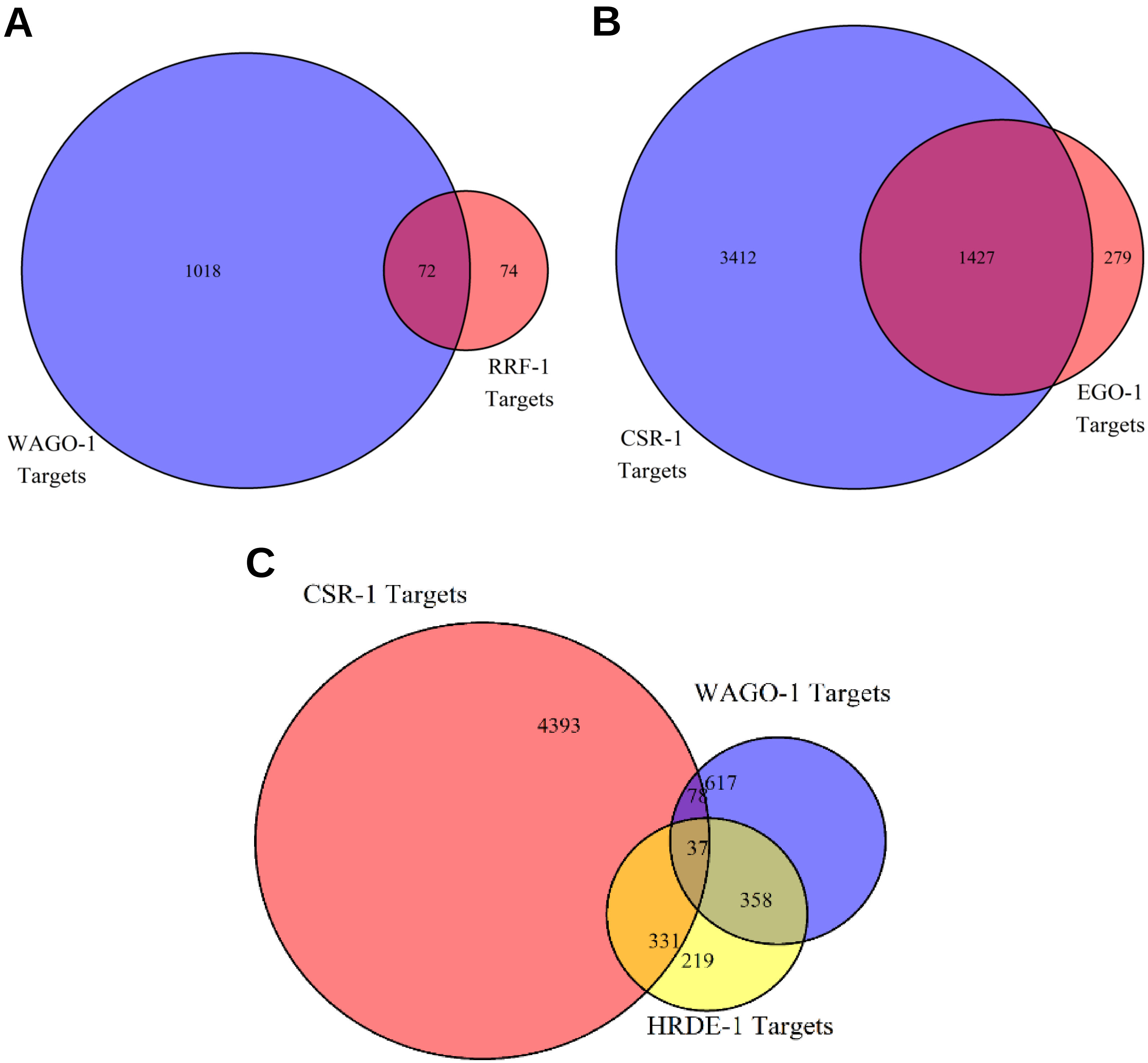
A
1018
72
74
RRF-1
Targets
WAGO-1
Targets
B
3412
1427
279
EGO-1
Targets
CSR-1
Targets
C
CSR-1 Targets
4393
WAGO-1 Targets
617
78
37
358
331
219
HRDE-1 Targets

Figure S12.

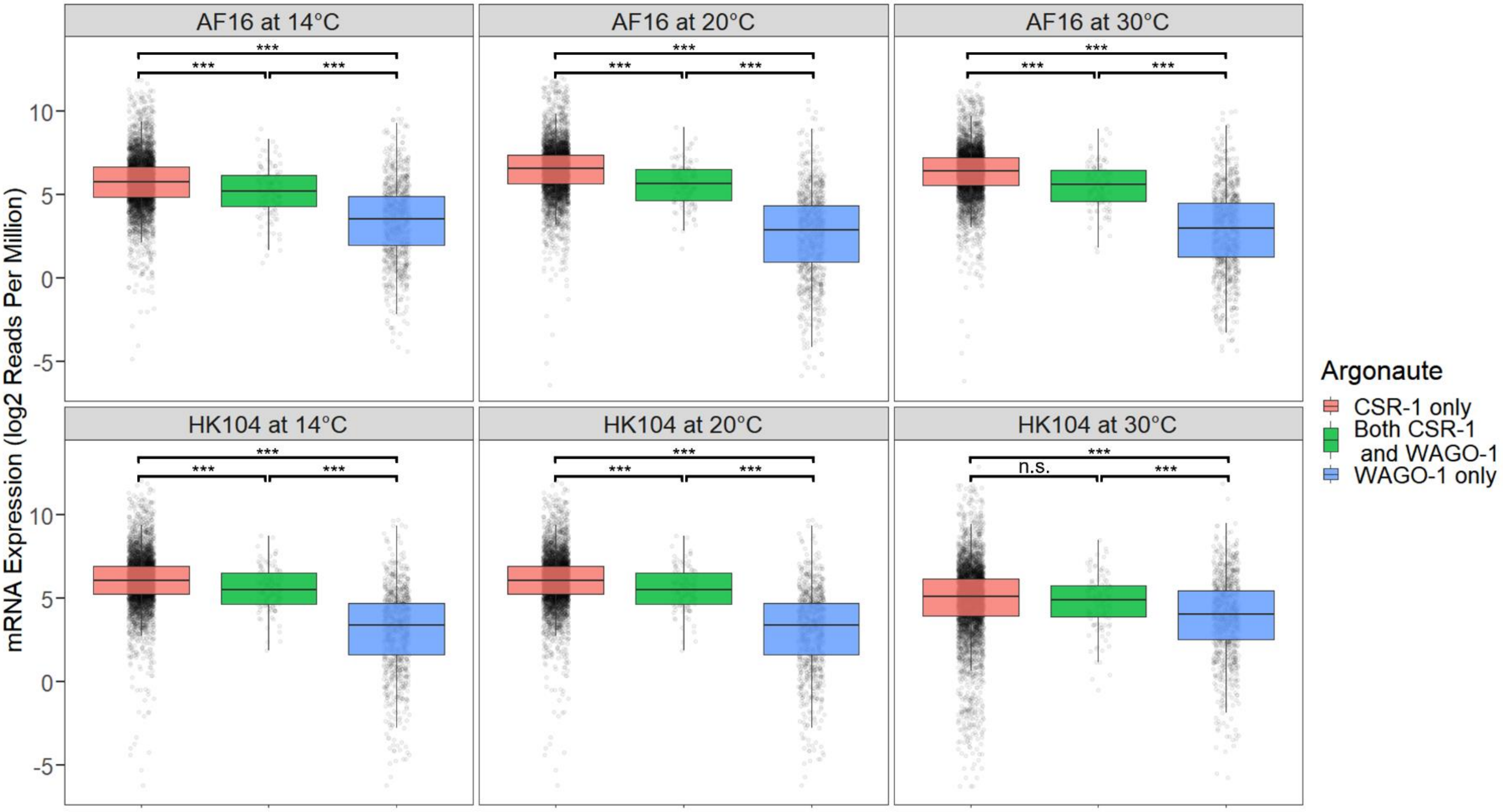
AF16 at 14°C
AF16 at 20°C
AF16 at 30°C
HK104 at 14°C
HK104 at 20°C
HK104 at 30°C
***
n.s.
mRNA Expression (log2 Reads Per Million)
10
5
0
-5
Argonaute
CSR-1 only
Both CSR-1 and WAGO-1
WAGO-1 only

Figure S13.

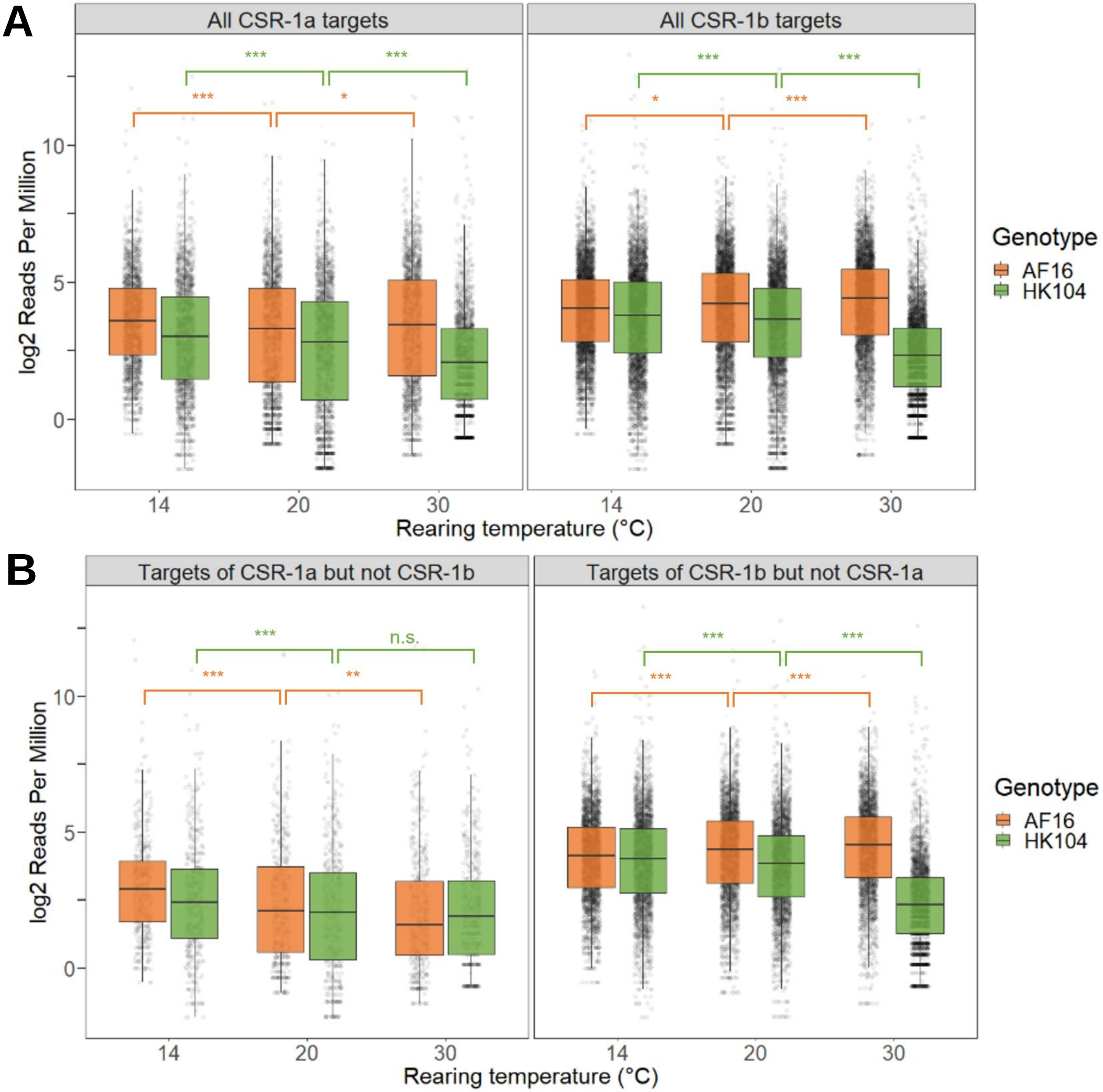

A
All CSR-1a targets
All CSR-1b targets
log2 Reads Per Million
Rearing temperature (°C)
Genotype
AF16
HK104
B
Targets of CSR-1a but not CSR-1b
Targets of CSR-1b but not CSR-1a
n.s.

Figure S14.

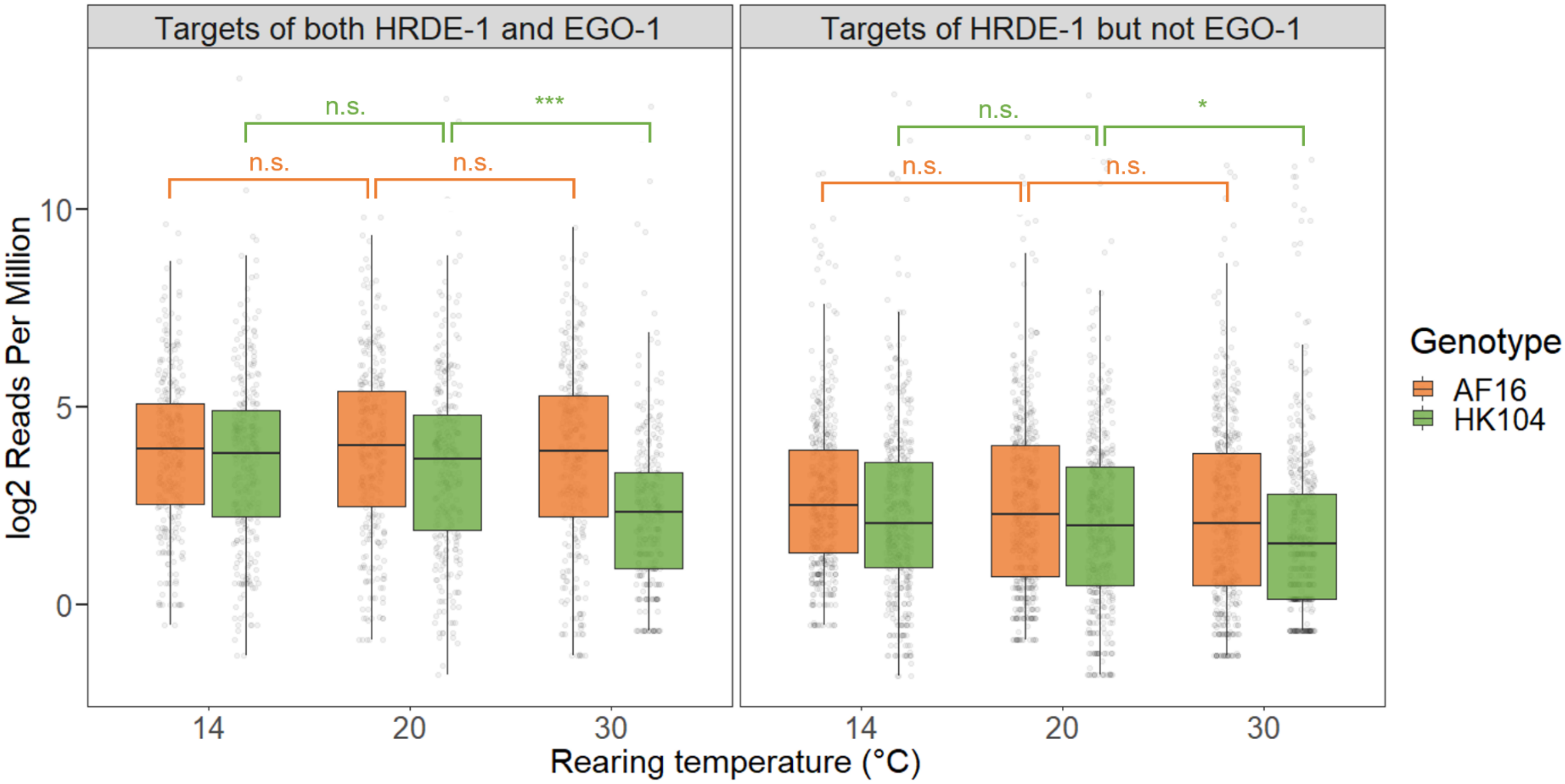
Targets of both HRDE-1 and EGO-1
Targets of HRDE-1 but not EGO-1
n.s.
***
n.s.
n.s.
n.s.
*
n.s.
n.s.
log2 Reads Per Million
10
5
0
14
20
30
14
20
30
Rearing temperature (°C)
Genotype
AF16
HK104

Figure S15.

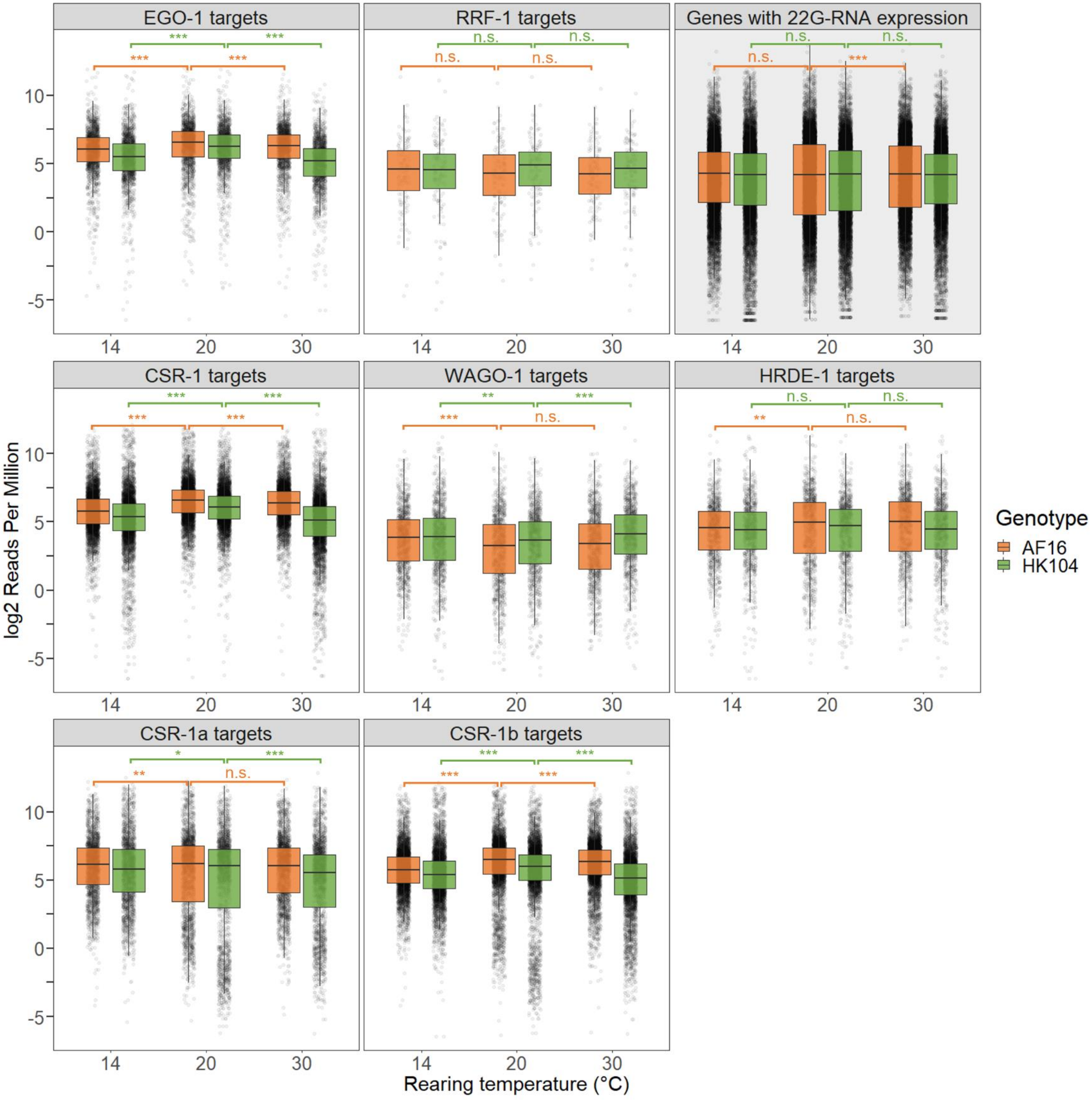
EGO-1 targets
RRF-1 targets
Genes with 22G-RNA expression
CSR-1 targets
WAGO-1 targets
HRDE-1 targets
CSR-1a targets
CSR-1b targets
log2 Reads Per Million
Rearing temperature (°C)
14
20
30
Genotype
AF16
HK104
n.s.

Figure S16.

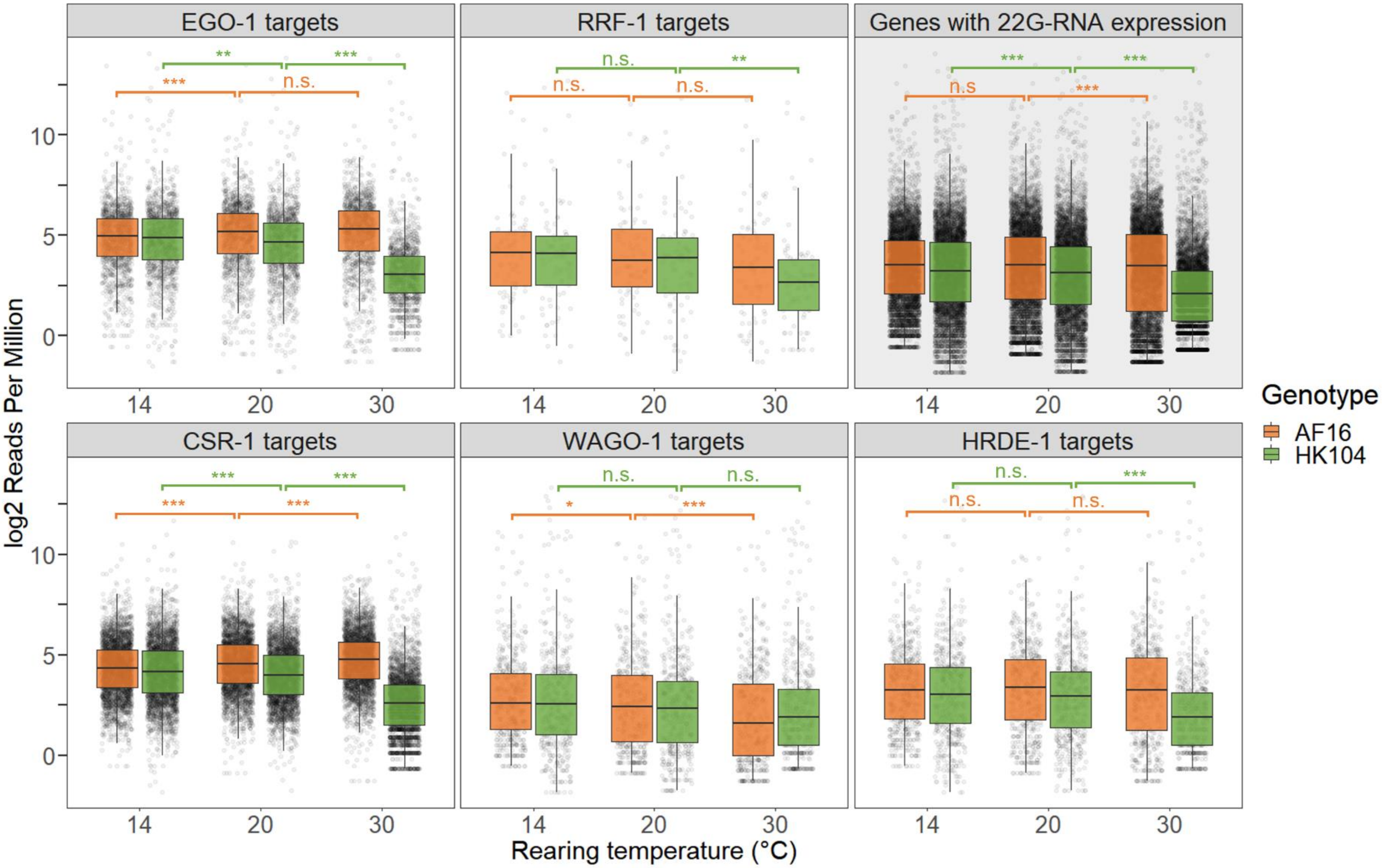

EGO-1 targets
RRF-1 targets
Genes with 22G-RNA expression
CSR-1 targets
WAGO-1 targets
HRDE-1 targets
log2 Reads Per Million
Rearing temperature (°C)
Genotype
AF16
HK104

Figure S17.

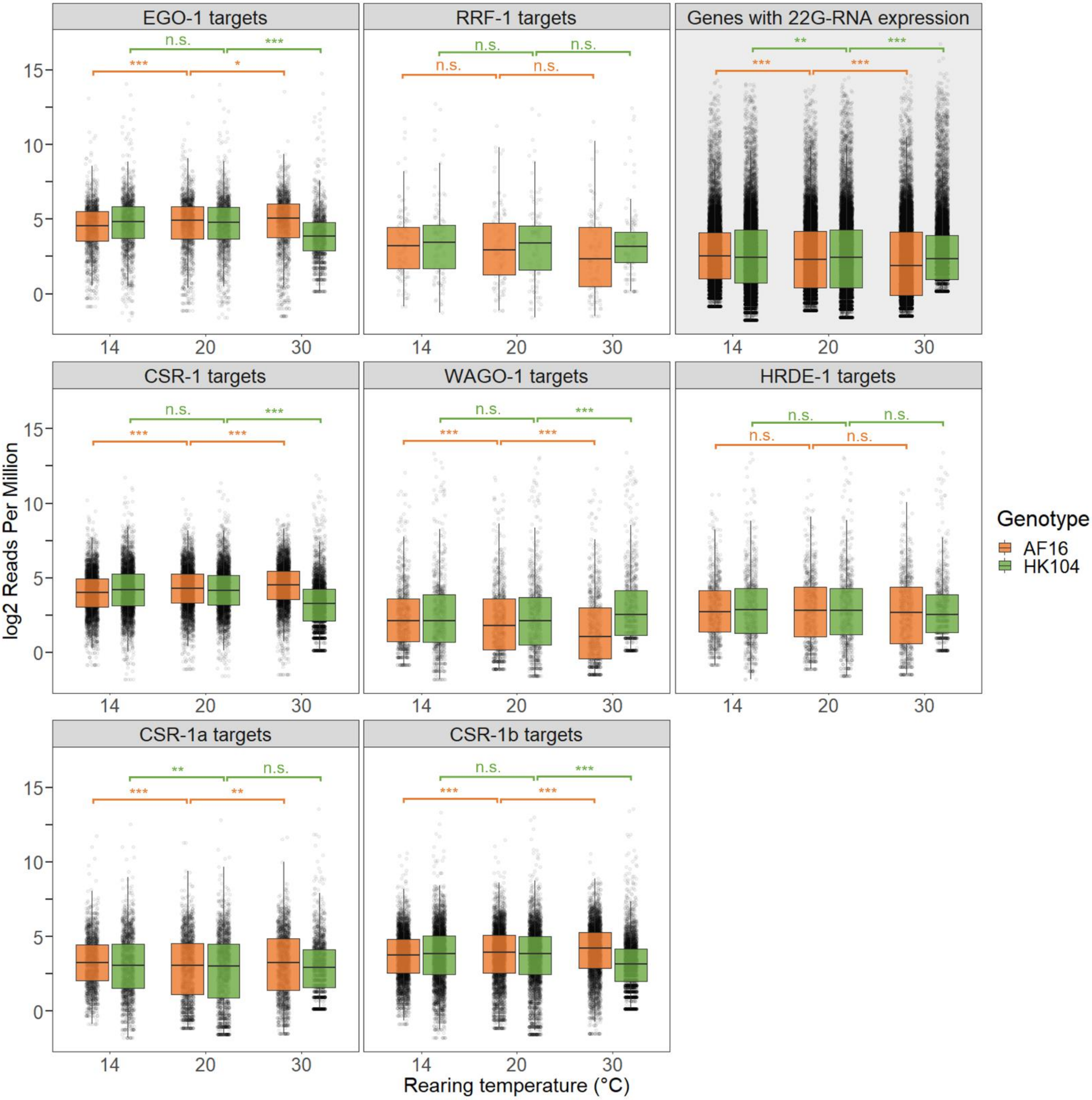

EGO-1 targets
RRF-1 targets
Genes with 22G-RNA expression
CSR-1 targets
WAGO-1 targets
HRDE-1 targets
CSR-1a targets
CSR-1b targets
log2 Reads Per Million
Rearing temperature (°C)
Genotype
AF16
HK104
